# The biodiversity hotspot as evolutionary hot-bed: spectacular radiation of *Erica* in the Cape Floristic Region

**DOI:** 10.1186/s12862-016-0764-3

**Published:** 2016-09-17

**Authors:** M. D. Pirie, E. G. H. Oliver, A. Mugrabi de Kuppler, B. Gehrke, N. C. Le Maitre, M. Kandziora, D. U. Bellstedt

**Affiliations:** 1 Department of Biochemistry, University of Stellenbosch, Private Bag X1, Matieland, 7602 South Africa; 2 Institut für Spezielle Botanik und Botanischer Garten, Johannes Gutenberg-Universität, Anselm-Franz-von-Bentzelweg 9a, 55099 Mainz, Germany; 3 Department of Botany and Zoology, University of Stellenbosch, Private Bag X1, Matieland, 7602 South Africa; 4 INRES Pflanzenzüchtung, Rheinische Friedrich-Wilhelms-Universität Bonn, Katzenburgweg 5, 53115 Bonn, Germany

**Keywords:** Biodiversity, Cape Floristic Region, Diversification, *Erica*, Evolution

## Abstract

**Background:**

The disproportionate species richness of the world’s biodiversity hotspots could be explained by low extinction (the evolutionary “museum”) and/or high speciation (the “hot-bed”) models. We test these models using the largest of the species rich plant groups that characterise the botanically diverse Cape Floristic Region (CFR): the genus *Erica* L. We generate a novel phylogenetic hypothesis informed by nuclear and plastid DNA sequences of c. 60 % of the c. 800 *Erica* species (of which 690 are endemic to the CFR), and use this to estimate clade ages (using RELTIME; BEAST), net diversification rates (GEIGER), and shifts in rates of diversification in different areas (BAMM; MuSSE).

**Results:**

The diversity of *Erica* species in the CFR is the result of a single radiation within the last c. 15 million years. Compared to ancestral lineages in the Palearctic, the rate of speciation accelerated across Africa and Madagascar, with a further burst of speciation within the CFR that also exceeds the net diversification rates of other Cape clades.

**Conclusions:**

*Erica* exemplifies the “hotbed” model of assemblage through recent speciation, implying that with the advent of the modern Cape a multitude of new niches opened and were successively occupied through local species diversification.

**Electronic supplementary material:**

The online version of this article (doi:10.1186/s12862-016-0764-3) contains supplementary material, which is available to authorized users.

## Background

Biological diversity is spread unevenly across the globe and across the tree of life, clustered in geographic hotspots [1] and species-rich clades [2–4]. Diverse organisms with a range of life history and other traits have radiated in environments with different topographies, climates, and histories. The hyper-diverse tropical Andes set the stage for a spectacular radiation of lupins (*Lupinus*; Fabaceae) [2], the Amazon rainforest for that of *Inga* (Fabaceae) [3], the Mediterranean hotspot for that of carnations (*Dianthus*; Caryophyllaceae) [4] and Southern Africa’s succulent karoo for that of ice plants (Ruschioideae; Aizoaceae) [5]. These species-rich groups present us with a rich and powerful source of data for bettering our understanding of the origins of biological diversity: we can analyse numerous speciation events in comparable biological systems within evolutionarily recent, and hence more tractable, timescales.

The mountainous landscape of South Africa’s Cape Floristic Region (CFR) hotspot [1] hosts 9000 plant species, 70 % endemic [6, 7], within only c. 90,000 km^2^. Thirty-three species rich “Cape clades” collectively account for around half of this remarkable richness [7], of which the genus *Erica* L. would be the largest, if the around 690 species [8] represent a single clade. *Erica* species are woody shrubs that dominate the CFR’s heathland “fynbos” vegetation as well as heathland ecosystems in the western Palearctic (including the Mediterranean) and mountain “sky islands” of Tropical Africa [9] and Madagascar. However, the numbers of species in regions outside the CFR are lower by an order of magnitude. Such striking regional asymmetries in species richness within a group of notably consistent habit pose a fascinating evolutionary conundrum, the solutions for which can inform our general understanding of the assemblage of regional biotas.

Here we ask: a) Does the extraordinary diversity of *Erica* in the CFR stem from a single common ancestor in the Cape? b) Are regional asymmetries in species richness the result of shifts in rates of diversification within the *Erica* clade and in different areas? c) Does the radiation of Cape *Erica* reflect a ‘museum’ (low extinction) and/or ‘hot-bed’ (high speciation) model for the biotic assemblage of the CFR? Such an analysis demands a credible, detailed and dated phylogenetic tree of the group: we present a phylogenetic hypothesis for *Erica* based on greatly increased sampling of species and molecular markers.

## Methods

Taxon and character sampling: Our phylogenetic hypothesis is informed by nuclear and plastid DNA sequences of c. 60 % of all *Erica* species, represented by 606 accessions of 488 species and 28 sub-specific taxa from across the geographic range of the clade (17 of 19 Palearctic species [89 %], 414 of 690 CFR [60 %]; 13 of 23 Tropical Africa [57 %]; 27 of 51 Drakensberg [53 %]; and 17 of c. 41 Madagascar/Mascarenes [42 %]), plus six outgroups (Additional file 1: Table S1). Specimens were collected in the field and determined by EGHO. Vouchers were lodged in herbaria (Additional file 1: Table S1), and leaf samples dried in silica gel and archived at -20 °C to preserve the DNA. Most sequences were obtained newly for this study, with some from previous work [10–12]. We obtained DNA sequences mostly using a direct PCR amplification protocol [13] with universal angiosperm primers [14, 15] as described in [12]. We employed a targeted supermatrix sampling strategy [16]: ITS and chloroplast *trnT-trnL* and *trnL-trnF-ndhJ* spacer sequences were obtained for all samples, and other plastid markers (*trnL* intron, *atpI-atpH* spacer, *trnK-matK* intron and *matK* gene, *psbM-trnH* spacer, *rbcL* gene, *rpl16* intron, *trnL-rpl32* spacer) were added for taxa selected, on the basis of preliminary analyses, as representative of early diverging lineages within each of the major subclades, in order to improve resolution of deeper nodes in the plastid tree. Sequences in general, and particularly ITS, were inspected to confirm the absence of polymorphism and (other) evidence of paralogy (e.g. indels in coding regions). An accessions table including Genbank accessions numbers is presented in Additional file 1: Table S1.

Phylogenetic inference: Individual matrices including all sequences for each marker were aligned in Mesquite [17] and imported into SequenceMatrix [18] to export concatenated matrices (excluding taxa causing topological conflict between gene trees; see below) for further analyses. A matrix of 63 phylogenetically representative taxa for which a minimum of 14 of the 20 data partitions were available was analysed using PartitionFinder [19] to infer best fitting data partitioning strategies and substitution models (heuristic search strategy ‘greedy’; comparison of fit using the Bayesian information criterion). Individual markers, coding and non-coding regions within those markers, and codon positions within protein coding genes were all specified as potential data partitions. Maximum likelihood (ML) analyses were performed using RAxML on CIPRES [20, 21] incorporating the data partitions inferred using PartitionFinder. Clade support was estimated using bootstrapping halted automatically by RAxML following the majority-rule ‘autoMRE’ criterion. To test for experimental error, confirm congruence of individual plastid markers, and to infer and compare gene trees we performed preliminary phylogenetic analyses of individual markers separately. These were followed by final analyses of ITS, combined plastid data and combined ITS and plastid data. Fifteen taxa causing topological conflict subject to ≥70 % bootstrap support (BS) between ITS and combined plastid gene trees were excluded from analyses of the concatenated data (leaving 597) under the assumption that such conflicts reflect (apparently uncommon) incidences of reticulation or incomplete lineage sorting that violate the assumption of a bifurcating tree [22]. Further phylogenetic analyses were performed using BEAST 1.8 [23] (as below).

Molecular dating: Two dating methods were employed on the Ericeae matrix: BEAST [23], using the 63 taxa matrix but excluding the most distant outgroup, *Empetrum*; and RELTIME [24], using the 597 taxa ML tree from the RAxML concatenated data analysis, removing *Empetrum* and *Corema album*. We used the 63 taxa matrix with BEAST because of the failure of multiple runs to converge with the full supermatrix, a not unexpected phenomenon in the presence of large proportions of missing data [16]. The targeted sampling strategy meant that the same internal focal nodes are represented in both trees. For BEAST, the root age (most recent common ancestor of *Erica* and *Daboecia*) was constrained based on the results of [25] in Ericaceae-wide analyses employing multiple fossil calibrations (producing results consistent with those presented in [26]). We used a normal distribution with mean 62 Mya and SD 10, giving a 95 % prior probability distribution of 42-82 Mya reflecting uncertainty in the original analyses [25]. In a further analysis an additional prior was implemented to reflect the age of Ericaceae pollen in sediments offshore of Southern Africa [27] and thereby test the impact on age estimates assuming that this pollen record represents *Erica*. For this, we used an exponential distribution with offset of 10 Mya (a hard minimum) and mean of 2.0, giving a 95 % prior probability distribution of 10-16 Mya (i.e. a soft maximum) for the stem node of Cape *Erica*. This is to assume that the Cape *Erica* clade is at least as old as the age of the pollen record and may be older to a limited degree. Following preliminary partitioned analyses that failed to converge, the data were not partitioned; we applied a GTR + G substitution model, lognormal relaxed clock, Yule process speciation model, and otherwise default priors, and performed two runs of 10 million generations sampling every 1000 in each case. Convergence was assessed using TRACER 1.6 [28] and Are We There Yet [29], and the results summarised using programs of the BEAST package. For RELTIME we assumed local clocks and imposed age constraints by means of a point estimate for the root node (the minimum, mean and maximum ages as above).

Diversification rates analyses: To infer the net diversification rate of the *Erica* Cape clade and compare it to those of other Cape and rapidly radiating clades, we used the method of Magallón & Sanderson ([30], as implemented in GEIGER; [31]). For Cape *Erica*, we used species richness and full range of crown node ages (minimum and maximum under RELTIME and highest posterior density intervals under BEAST) as inferred here. For comparison, we performed the same calculations based on data from the literature for the recent rapid radiations of lupins [2], *Inga* [3], carnations [4], and ice plants [5]; as well as the Cape clades *Muraltia* [32], *Pentameris* [33] *Protea* [34] and Restionoideae (“African Restionaceae”) [32]. The latter are examples for which detailed time-calibrated phylogenies of ancestrally CFR species – not those that also diversified in other areas – are available. We did not account for the impact on crown node age estimates of unsampled species during the calculation, and used relative extinction rates of 0.9 and zero across the board.

**Fig. 1:**
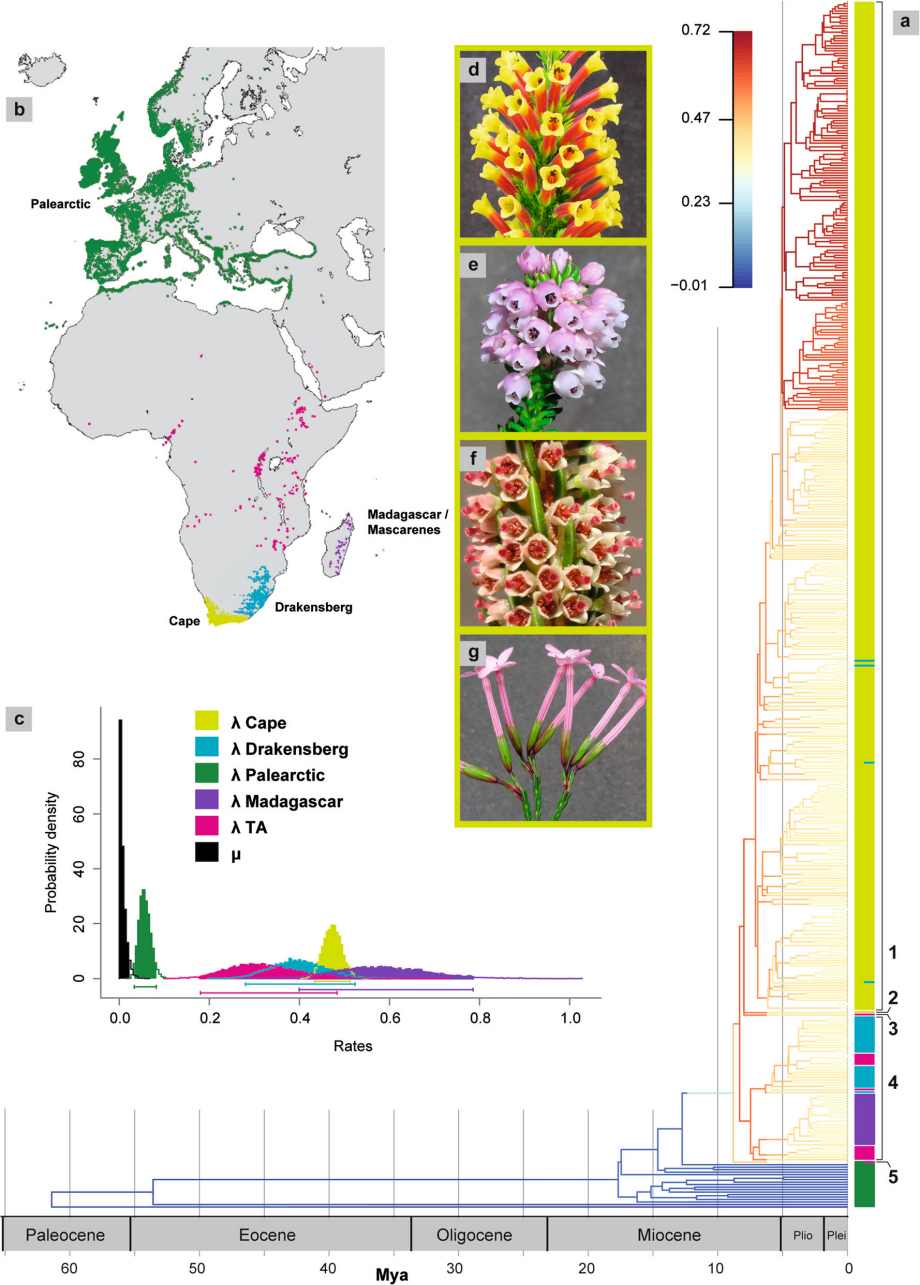
The diversification of *Erica* in space and time. **a** Time-calibrated phylogenetic tree of 478 extant lineages that populated the radiation of *Erica* with branches coloured according to mean net diversification rates (scale indicates species per million years) inferred using BAMM, with regions of samples indicated by the coloured bar at the terminals and clades/species referred to in the text indicated with numbers: 1 = Cape clade; 2 = *E. pauciovulata*; 3 = *E. trimera*; 4 = Afrotemperate clade; 5 = *E. arborea*. **b** Geographic distribution of *Erica*, based on collections databased by GBIF, showing Palearctic, Tropical Africa, Madagascar, Drakensberg and Cape regions. **c** Region specific speciation rates (λ) and the single extinction rate (μ). **d**-**g** Examples of the spectacular floral diversity of Cape *Erica*: d) *E. macowanii*, e) *E. pulvinata*, f) *E. coarctata*, and g) *E. jasminiflora*

To test whether diversification rate heterogeneity reflects different speciation and extinction rates between geographic areas, we used MuSSE (Multiple State Speciation and Extinction) as implemented in diversitree 0.93 [35]. MuSSE uses maximum likelihood to estimate the values of different parameters under a constant birth death model: speciation (λ) and extinction (μ) rates under each of the discrete states of the character (in this case, geographic distribution), and rates of transition (q) from one state (area) to another. We compared the rates between Palearctic, Tropical African, Madagascan, Drakensberg and Cape species of *Erica*, *Calluna* and *Daboecia*. The areas are indicated in Fig. 1 and were so defined because they are often compared in the literature, are largely geographically isolated and <1 % of *Erica* species are widespread between any two of them (these limited to two species in both the Cape and Drakensberg – *E. caffra* and *E. cerinthoides* – and one in the Palearctic and Tropical Africa – *E. arborea*). We used the discrete multistate model, instead of GeoSSE, that models widespread geographic distributions, to represent multiple areas (rather than just two in GeoSSE) under the assumption that widespread distributions were rare throughout the evolutionary history of the group. We coded the three samples of widespread species according to the region in which they were collected under the assumption that effectively failing to sample such species across their wider distribution would have little impact on the results. We used the rate-smoothed 597-taxa RAxML tree, having removed multiple accessions of species and outgroups (leaving 487 terminals), and corrected for incomplete sampling by assigning region-specific sampling fractions. We did not consider phylogenetic uncertainty, as the major clades are well supported and largely restricted to single regions and thus the uncertainty regarding our question remains low. We compared maximum likelihood estimates given models considering different regions (either 5 distinct regions or combinations of Palearctic, Cape and the rest of Africa or of Europe versus Africa and or Cape versus other regions) and considering single versus multiple rates for speciation and/or extinction. For all but the unconstrained model, we constrained the transition rates to one parameter. Thereafter, for the best model, we tested whether constraining the transition rate reduces the likelihood. We compared the fit of the models to the data using the anova function in diversitree and using the AIC to compare the fit of the models. The parameters for the best fitting model were then calculated using a Bayesian MCMC approach run for 10,000 steps using an exponential probability distribution as prior for the underlying rates in the model. We assessed convergence by comparing the probability values of the sampling after excluding a burnin of 25 %.

To further determine whether there is diversification rate heterogeneity in the *Erica* dataset, we used BAMM 2.5 and Bammtools 2.1 [36, 37]. The method compares the fit of different models (a series of diversification processes) assuming different numbers of shifts based on a reversible jump MCMC to explore parameter space. We used the pruned, rate-smoothed RAxML tree, as above, and corrected for non-random species sampling by assigning regional specific proportions to the few, largely endemic, clades. We used “setBAMMpriors” to adjust the priors according to the scaling of the tree. The initial speciation rate was set to 0.18 and extinction rate to 0.111 according to inferred rates for Ericaceae [25]. Preliminary results showed that different initial speciation and extinction rate did not have a large effect on our results. The MCMC was run for 10,000,000 generations, with every 1000 generation saved. To assess convergence, the likelihood of all sampled generations was plotted in R (burnin = 10 %) and ESS values for the likelihood and the inferred numbers of shifts were calculated using the coda package [38]. It was not possible to compare Bayes Factors for zero rate shifts with those for given numbers of shifts (see BAMM Documentation part 7.6), but we compared the prior probability of a given number of shifts to the posterior probability to confirm that these differed. We then computed the set of credible shifts and reconstructed the mean of the marginal posterior density of speciation, extinction and net diversification rates across the tree. We sought to assess whether the BAMM results are dependent on the particular topology and branch lengths of the phylogenetic tree used above by repeating the analyses with 25 randomly selected, rate smoothed and pruned RAxML bootstrap trees.

## Results

Results of preliminary analyses of individual plastid markers (TreeBase study accession URL: http://purl.org/phylo/treebase/phylows/study/TB2:S18291) showed no conflicting nodes supported at ≥70 % bootstrap support (BS). The better resolved combined plastid gene tree (Additional file 2: Figure S1a) was largely consistent with that of ITS (Additional file 2: Figure S1b). Fifteen taxa causing topological conflict subject to ≥70 % BS between ITS and combined plastid gene trees (indicated in Additional file 2: Figures S1a and b) were excluded from analyses of the concatenated data (leaving 597; Additional file 2: Figure S1c). Further exclusion of one Cape species, *E. pauciovulata*, resulted in an increase in support for a single Cape clade (see below) from 70 % BS (Additional file 2: Figure S1c) to 89 % (Additional file 2: Figure S1d).

The *Erica* phylogeny roots in a northern Palearctic grade subtending a southern African/Madagascan clade. The latter comprises a deep polytomy including a) the Cape clade, including all but one Cape species plus four found in the Drakensberg (two of which also distributed in the Cape), b) a single further Cape species, *E. pauciovulata*, c) *E. trimera* (Tropical Africa), d) the ‘Extra-CFR African clade’ that includes all other Drakensberg and Tropical African species (except for *E. arborea*) and a clade of all Madagascan/Mascarene species, and e) *E. arborea* (Palearctic and TA) (Fig. 1, Additional file 2: Figure S1). Our age estimates for clades within *Erica* (Fig. 1, Additional file 3: Figure S2a-c) are based on the similar results of both the two relaxed clock molecular dating methods (RELTIME, Additional file 3: Figure S2a; and BEAST, Additional file 3: Figure S2b) with secondary calibration, and additional BEAST results (Additional file 3: Figure S2c) further constrained using the Southern Africa offshore microfossil record. The crown node of *Erica* was estimated at 18 (24-12) Mya (RELTIME on the matrix of 597 taxa; Additional file 3: Figure S2a) and 27-19/31-12 Mya (95 % posterior probability ranges from BEAST, using the reduced matrix of 62 taxa, with/without microfossil evidence; Additional file 3: Figure S2b/c). The radiation of lineages within the African/Madagascan clade was estimated at 9 (12-6) Mya (RELTIME) and 14-11/17-7 Mya (BEAST). The stem node of the Cape clade was estimated at 8 (11-6) Mya (RELTIME) and 12-10/15-6 Mya (BEAST); the crown node at 7 (9-5) Mya (RELTIME) and 11-9/15-6 Mya (BEAST).

Given our dated phylogenetic trees, the net diversification rate of Cape *Erica* was 0.28-0.7 (assuming relative extinction of 0.9) or 0.39-0.97 (relative extinction zero) species per million years; estimated rates of other Cape clades and faster recent species radiations reported worldwide are presented in Table 1.

**Table 1:** Plant diversification rates in the CFR and beyond. Net diversification rates of Cape clades and other recent radiations worldwide in species per million years, estimated using species numbers and clades ages with the method of S Magallón and MJ Sanderson [30]

Clade	Species numbers	Crown age (Mya)	Reference	Rate (Species/Mya) relative extinctio*n* = 0.9	Rate (Species/Mya) relative extinctio*n* = 0.0	Note
Cape clades:						
Cape *Erica*	690	6.0–15.0	This paper	0.28–0.70	0.39–0.97	Range of estimates from RELTIME and BEAST analyses
*Muraltia*	124	8.6–16.4	[32]	0.15–0.29	0.25–0.48	Presented range (molecular dating)
Restionoideae	350	31.7–65.4	[32]	0.05–0.11	0.07–0.16	Presented range (molecular dating)
*Pentameris*	83	13.2–16.1	[33]	0.13–0.16	0.23–0.28	Presented range (molecular dating)
Protea	69	11.2–27.2	[34]	0.07–0.18	0.13–0.32	Presented range (molecular dating)
Other clades:						
Andean lupins	85	1.6–2.3	[2]	0.96–1.38	1.64–2.37	*Lupinus* stem calibrated at 21.16 Ma
		1.2–1.8	As above	1.24–1.86	2.13–3.18	*Lupinus* stem calibrated at 16.01 Ma
Ice plants (Aizoaceae: Ruschioideae)	1563	0.6–7.0	[5]	0.28–3.32	0.5–5.88	Calibrated with ITS substitution rates
		8.0–9.4	As above	0.53–0.63	0.71–0.83	Calibrated with plastid substitution rates
European *Dianthus*	200	0.61–2.4	[4]	1.23–4.90	1.9–7.55	Min. and max. ages reported
*Inga*	300	1.6–9.8	[3]	0.34–2.11	0.51–3.13	Calibrated with *trnL-F*/ITS substitution rates

MuSSE analyses performed with diversitree identified differences in speciation rates specific to geographic regions, with the best scoring model otherwise including only single rates for extinction and for transitions (dispersals between regions; Table 2). The lowest diversification rate is in the Palearctic, while rates in all other regions are high (Fig. 1c; Table 3). Three further models scored within ≤2 of the best model according to the AIC (Table 2); these included single rates for transitions, either five or two parameters for region-specific speciation rates (the latter, Palearctic versus Africa) and two differing rates for extinction (either Palearctic versus Africa or Cape versus all other areas; Additional file 4: Table S2). The inferred rates for extinction were universally similar and low (Additional file 4: Table S2)

**Table 2:** Comparison of different MuSSE models estimated for 478 species of *Erica*

Geographical regions	constraints	No. of λ parameters	No. of μ parameters	No. of q parameters	lnLik	AIC	AIC weights	Parameter estimates
Palearctic, Cape, Drakensberg, Madagascar, Tropical Africa	-	5	5	20	-1048.9	2157.7	6.056120e-06	
	λ, q	1	5	1	-1082.9	2179.8	9.621447e-11	
	***μ, q***	***5***	***1***	***1***	***-1060.8***	***2135.5***	***4.007405e-01***	Table 3
	μ	5	1	20	-1051.3	2154.7	2.714164e-05	
One region	λ, μ, q	1	1	1	-1162.5	2331.0	6.040484e-05	
Palearctic, Cape, rest of Africa	λ, q	3	5	1	-1062.4	2142.8	1.041570e-02	
	μ, q	5	3	1	-1060.8	2139.5	5.423432e-02	
	λ, μ, q	3	3	1	-1063.3	2140.6	3.129048e-02	
Palearctic, Africa	λ, q	2	5	1	-1062.4	2140.8	2.831280e-02	
	**μ, q**	**5**	**2**	**1**	**-1060.8**	**2137.5**	**1.474242e-01**	Additional file 4: Table S2, Model 1
	**λ, μ, q**	**2**	**2**	**1**	**-1063.6**	**2137.1**	**1.800643e-01**	Additional file 4: Table S2, Model 2
Cape, other	λ, q	2	5	1	-1081.1	2178.1	2.251079e-10	
	**μ, q**	**5**	**2**	**1**	**-1060.8**	**2137.5**	**1.474242e-01**	Additional file 4: Table S2, Model 3
	λ, μ, q	2	2	1	-1100.9	2211.7	1.138265e-17	

*Abbreviations*: λ – speciation rate, μ - extinction rate, q – transition rate, lnLik – logarithm of likelihood, AIC – Akaike information criterion. The best scoring model is indicated with bold italics (parameter estimates presented in Table 3); three models with AIC scores within 2 of the best scoring model are indicated in bold (parameter estimates presented in Additional file 4: Table S2)

**Table 3:** Parameter estimates given the best scoring MuSSE model

	λ Cape	λ Drakensberg	λ Palearctic	λ Madagascar	λ TA	μ	transition rate	*p*
Min.	0.4043	0.1971	0.01685	0.2771	0.1013	2.20E-7	2.39E-4	-1074
1st Qu.	0.4595	0.3575	0.04795	0.5179	0.2658	2.24E-3	1.15E-3	-1064
Median	0.4734	0.3978	0.05598	0.5818	0.3149	5.37E-3	1.43E-3	-1062
Mean	0.4735	0.4002	0.0569	0.5869	0.3216	7.65E-3	1.49E-3	-1062
3rd Qu.	0.4872	0.4402	0.06498	0.6527	0.3702	1.08E-2	1.77E-3	-1061
Max.	0.5536	0.6644	0.11022	1.0282	0.7366	6.40E-2	3.85E-3	-1059

**Fig. 2:**
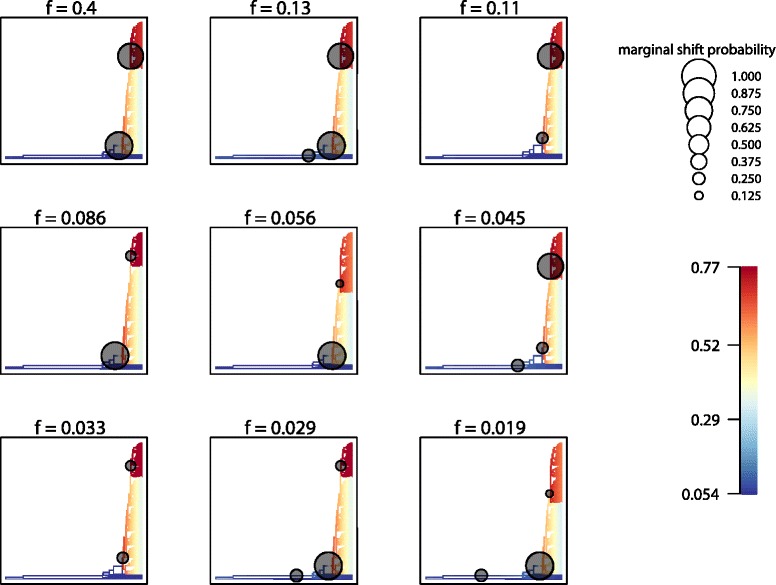
BAMM diversification rate results. Visualisations of the nine shift configurations from BAMM analysis with highest PP with branches coloured according to mean net diversification rates (scale bar indicates species per million years)

BAMM analyses also indicated strong support for heterogeneous diversification dynamics within *Erica*, in the form of multiple accelerations in the rate of diversification (Figs. 1 and 2, Additional file 5: Figure S3a; posterior probability [PP] of a single rate model = 0; PP density of 2-4 rate shifts = 0.74; 2-5 rate shifts = 0.87; Additional file 5: Figure S3b). Extinction rates appear to be constant through time, but speciation rates vary greatly. We inferred 14 distinct configurations within the 95 % credible shift sets. Distinct diversification regimes were associated with the *Erica* clade, the African/Madagascan clade (either including *E. arborea*, or not) and within the Cape clade; the former is found in only four of the nine configurations with highest PP which together sum to PP > 0.90 (Fig. 2) (and generally fewer than half of each of the individual configurations based on 25 bootstrap trees), whilst the two latter shifts are found in all of them (the African/Madagascan clade shift in almost all, and the shift within the Cape clade generally in more than half of the individual bootstrap configurations; Additional file 6: Table S3).

## Discussion

Whilst the richness of *Erica* species in the CFR is renowned, our results can finally confirm *Erica* as the most species rich Cape clade. With the possible exception of a single anomalous species (*E. pauciovulata*), all *Erica* in the CFR that we analysed can be traced back to a single common ancestor that colonised the region no earlier than c. 15 Mya, and all but a handful are endemic (Fig. 1). A Late Miocene initiation of the *Erica* radiation in the CFR is consistent with the first appearance of pollen of Ericaceae (and various other typical fynbos groups) in the fossil record in Southern Africa after 10 Mya [27]. Cape clades differ widely in age [39], and Cape *Erica* is neither conspicuously old nor young in this context. Its net diversification rate is modest compared to the most spectacular examples in flowering plants, documented from the much greater areas of the Andean Páramo [2] and the Mediterranean [4]. The *Erica* diversification rate is more similar to those of other rapidly evolving Cape clades, although notably faster than any that we compared (Table 1).

This remarkable radiation of *Erica* in the CFR is in stark contrast to the comparatively impoverished older Palearctic lineages. The heathers originated in the Northern Hemisphere [10, 12] and northern lineages (including monotypic *Calluna* and two species of *Daboecia*) are older than the single southern *Erica* clade (‘African/Malagasy Erica’; [10]). Higher diversity in Cape compared to Mediterranean clades has been attributed to lower rates of extinction [40]. Our results instead imply slower speciation in the wider western Palearctic (Fig. 1; Table 3 and Additional file 4: Table S2), although this conclusion must be qualified by the known difficulty of inferring extinction rates from molecular phylogenies [41]. Although the ranges of speciation (and hence net diversification) rates in different regions outside the Palearctic overlapped (perhaps a methodological artefact caused by the much lower species numbers outside the Cape [42]), we discovered evidence for a rate increase within the Cape clade (Figs. 1 and 2). Phylogenetic uncertainty within the Cape clade is considerable (reflecting the short internal branches typical of bursts of lineage diversification [37]), but geographically, this diversification centres on lineages of the large SW-clade [10] mostly restricted within the South-Western CFR. Irrespective of inferred shifts in diversification rates, the greater areas of equivalent habitat in Tropical Africa and the Drakensberg (for similarly distributed *Protea*, estimated at roughly 17-fold; [34]) and in Madagascar compared to the CFR represent far lower densities of species and of speciation events through time given the phylogeny and clade ages inferred here.

The CFR is one of a number of mountainous and Mediterranean climate regions with unique and hyper-diverse biotas that both coincidentally, and as the result of worldwide climatic changes, originated within similar, relatively recent timeframes [2, 25, 40, 43]. The modern CFR was shaped by globally influenced palaeoclimatic dynamics that established during the Miocene, particularly world-wide cooling that led to aridification [44], and the establishment of the cold Benguela current off the south-west African coast, that led to the development of a winter rainfall regime and frequent fires [45, 46]. The disappearance of more mesic tropical forest elements from fossil deposits [27, 47] was followed by the appearance of more arid and/or fire adapted elements such as Aizoaceae, including Ruschioideae (in the succulent karoo), and *Erica* (in the CFR) [27]; the latter with its reduced leaf area and resistant yet inflammable wax-rich cuticles [48] combined with post-fire re-sprouting and smoke-stimulated re-seeding recruitment strategies [49, 50].

As with other mountainous hotspots [43], the CFR was also influenced by local uplift, that occurred during the Miocene [51, 52]. The high species richness and local endemism of the present day Cape is plausibly a direct result of this uplift: new niches opened, with physical barriers to gene flow between them, creating a stimulus for allopatric speciation [53]. Topographical complexity also creates local temperature and moisture gradients [53], and the patchwork of soils derived from the different lithologies of the Cape [54, 55] adds a further dimension to the resulting fine-scaled mosaic of habitats. By contrast to regions of the Northern Hemisphere, the Cape was buffered from the extremes of Pleistocene glacial cycles, and by implication from resulting extinction [56]; instead (less extreme) shifts in multiple local-scale ecological gradients, acting in concert, might actually drive speciation [57]. Key innovations in particular groups are also often mooted, such as adaptations to specialised pollinator interactions [58] that might reinforce speciation [59]. The numerous apparent shifts in pollination syndrome in *Erica* and the higher diversity of different syndromes in *Erica* in the Cape than elsewhere [10] make the latter a tempting explanation for the acceleration of the Cape *Erica* radiation.

However, meta-analyses of Cape phylogenies have provided support for multiple such hypotheses, with evidence for both ecological and/or pollinator shifts [60] and distributional and phenological shifts [61] in e.g. *Muraltia*, Cape Restionaceae and *Pentameris*; each of these and others too (such as edaphic shifts apparent in *Babiana* (Iridaceae) [62]) may have played a role. Given the results presented here, it is also plausible that (combinations of) factors specific to the most species-rich SW region may be responsible for the highest rates of diversification within the CFR. The relative contribution of these different factors overall is still hotly debated, and with a phylogenetic hypothesis for the clade now available, Cape *Erica* offers the greatest single source of data for further testing their importance in the assemblage of the flora.

## Conclusions

In two contrasting perspectives, the CFR is interpreted as a ‘museum’ of diversity [63], with persistence of pre-Miocene lineages [64] and lower extinction e.g. compared to the Mediterranean [40]; or the evolutionary ‘hot-bed’ of (recent) radiations [32, 39, 65, 66]. These models are not mutually exclusive [39, 67]. However, our results further weigh the balance in favour of the latter. The largest Cape clade, *Erica*, represents more species than included in most meta-analyses of Cape clades performed to date. Much of this remarkable diversity originated within the last few million years.

## Additional files

Additional file 1: Table S1. Accessions table including Genbank accession numbers. (XLSX 119 kb) Additional file 2: Figure S1. Phylogenetic hypotheses: best trees with bootstrap support values from RAxML analyses of a) concatenated plastid data and b) from nuclear ribosomal ITS (with taxa showing conflicting positions according to the two gene trees highlighted in yellow); and c) and d) of the combined data (excluding conflicting taxa): c) with and d) without *Erica pauciovulata* (exclusion of which leads to increased support for the Cape clade from 70 % to 89 %). (ZIP 8409 kb) Additional file 3: Figure S2. Relaxed clock molecular dating results: a) age estimates for clades within *Erica* inferred using RELTIME [24] with the best tree from RAxML (Additional file 2: Figure S1c); b) and c) phylogeny and relaxed clock molecular dating age estimates for clades within *Erica* inferred using BEAST [23] on a reduced matrix of 62 taxa b) with and c) without additional constraint based on microfossil evidence (error bars represent 95 % Posterior Probability (PP) intervals; PP clade support is indicated at nodes). (ZIP 11562 kb) Additional file 4: Table S2. Parameter estimates given the three best scoring suboptimal MuSSE models. (DOCX 15 kb) Additional file 5: Figures S3. BAMM diversification rate results: a) BAMM tree as presented in Fig. 1, including labels for tips and nodes referred to in the text; branches subtending *Erica*, *Calluna* and *Daboecia* are not to scale. b) Probabilities of overall numbers of diversification shifts inferred using BAMM. (ZIP 2444 kb) Additional file 6: Table S3. Summary of BAMM results based on 25 rate-smoothed RAxML bootstrap trees. (DOCX 12 kb)
