# Supplementary material for: The biodiversity hotspot as evolutionary hot-bed: spectacular radiation of Erica in the Cape Floristic Region
Source: BMC Evol Biol. 2016 Sep 17;16:190. doi: 10.1186/s12862-016-0764-3 (PMC5027107; doi:10.1186/s12862-016-0764-3)
Supplement: Additional file 2: Figure S1. — Phylogenetic hypotheses: best trees with bootstrap support values from RAxML analyses of a) concatenated plastid data and b) from nuclear ribosomal ITS (with taxa showing conflicting positions according to the two gene trees highlighted in yellow); and c) and d) of the combined data (excluding conflicting taxa): c) with and d) without Erica pauciovulata (exclusion of which leads to increased support for the Cape clade from 70 % to 89 %). (ZIP 8409 kb) [file 12862_2016_764_MOESM2_ESM.zip › add 4/S1a_Figure_RAxML_plastid_NEW.pdf]

# Figure S1a

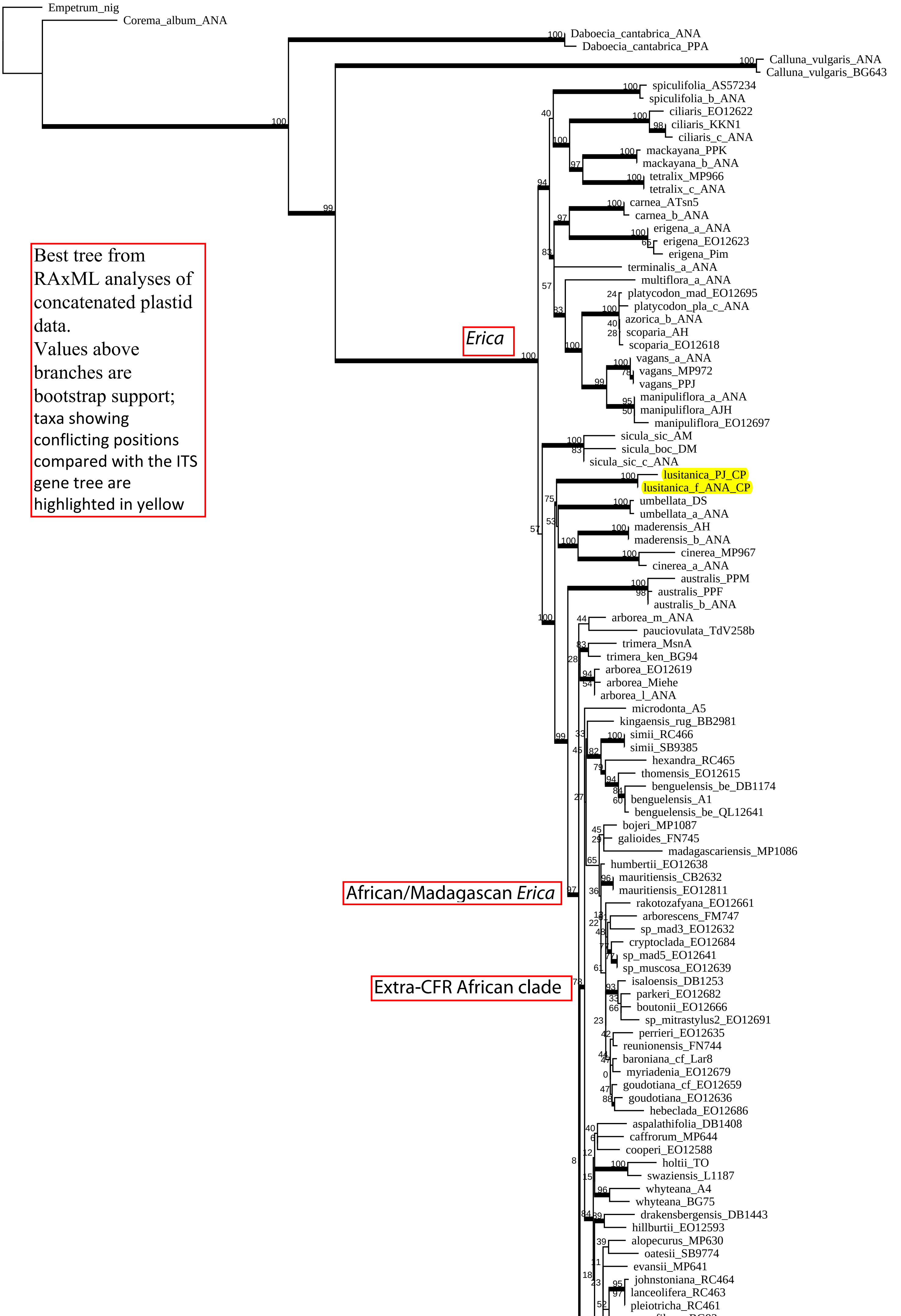


Best tree from RAxML analyses of concatenated plastid data.
Values above branches are bootstrap support; taxa showing conflicting positions compared with the ITS gene tree are highlighted in yellow

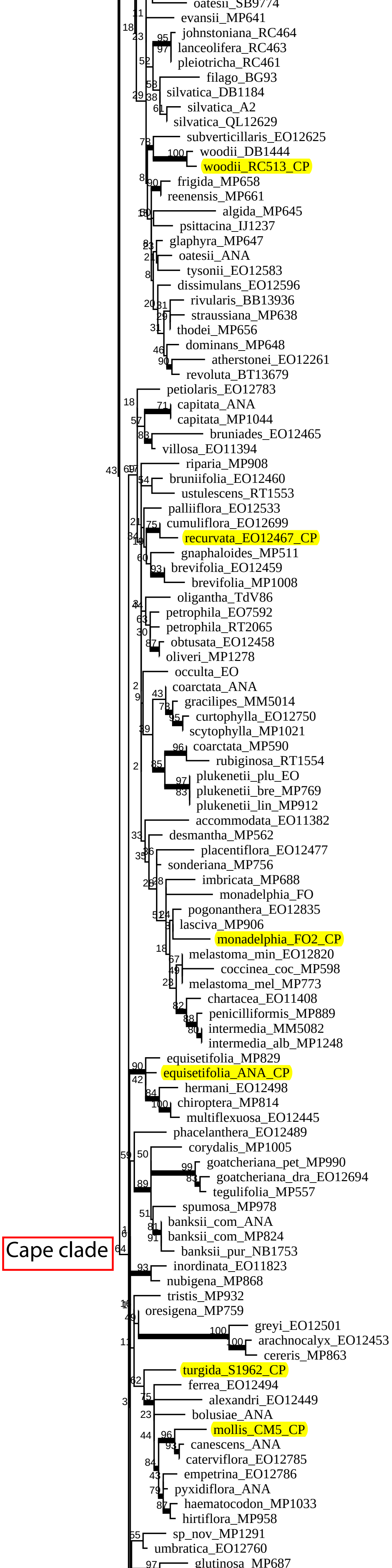

oatesii_SB9774
evansii_MP641
johnstoniana_RC464
lanceolifera_RC463
pleiotricha_RC461
filago_BG93
silvatica_DB1184
silvatica_A2
silvatica_QL12629
subverticillaris_EO12625
woodii_DB1444
woodii_RC513_CP
frigida_MP658
reenensis_MP661
algida_MP645
psittacina_IJ1237
glaphyra_MP647
oatesii_ANA
tysonii_EO12583
dissimulans_EO12596
rivularis_BB13936
straussiana_MP638
thodei_MP656
dominans_MP648
atherstonei_EO12261
revoluta_BT13679
petiolaris_EO12783
capitata_ANA
capitata_MP1044
bruniades_EO12465
villosa_EO11394
riparia_MP908
bruniifolia_EO12460
ustulescens_RT1553
palliiflora_EO12533
cumuliflora_EO12699
recurvata_EO12467_CP
gnaphaloides_MP511
brevifolia_EO12459
brevifolia_MP1008
oligantha_TdV86
petrophila_EO7592
petrophila_RT2065
obtusata_EO12458
oliveri_MP1278
occulta_EO
coarctata_ANA
gracilipes_MM5014
curtophylla_EO12750
scytophylla_MP1021
coarctata_MP590
rubiginosa_RT1554
plukenetii_plu_EO
plukenetii_bre_MP769
plukenetii_lin_MP912
accommodata_EO11382
desmantha_MP562
placentiflora_EO12477
sonderiana_MP756
imbricata_MP688
monadelphia_FO
pogonanthera_EO12835
lasciva_MP906
monadelphia_FO2_CP
melastoma_min_EO12820
coccinea_coc_MP598
melastoma_mel_MP773
chartacea_EO11408
penicilliformis_MP889
intermedia_MM5082
intermedia_alb_MP1248
equisetifolia_MP829
equisetifolia_ANA_CP
hermani_EO12498
chiroptera_MP814
multiflexuosa_EO12445
phacelanthera_EO12489
corydalis_MP1005
goatcheriana_pet_MP990
goatcheriana_dra_EO12694
tegulifolia_MP557
spumosa_MP978
banksii_com_ANA
banksii_com_MP824
banksii_pur_NB1753
Cape clade
inordinata_EO11823
nubigena_MP868
tristis_MP932
oresigena_MP759
greyi_EO12501
arachnocalyx_EO12453
cereris_MP863
turgida_S1962_CP
ferrea_EO12494
alexandri_EO12449
bolusiae_ANA
mollis_CM5_CP
canescens_ANA
caterviflora_EO12785
empetrina_EO12786
pyxidiflora_ANA
haematocodon_MP1033
hirtiflora_MP958
sp_nov_MP1291
umbratica_EO12760
glutinosa_MP687

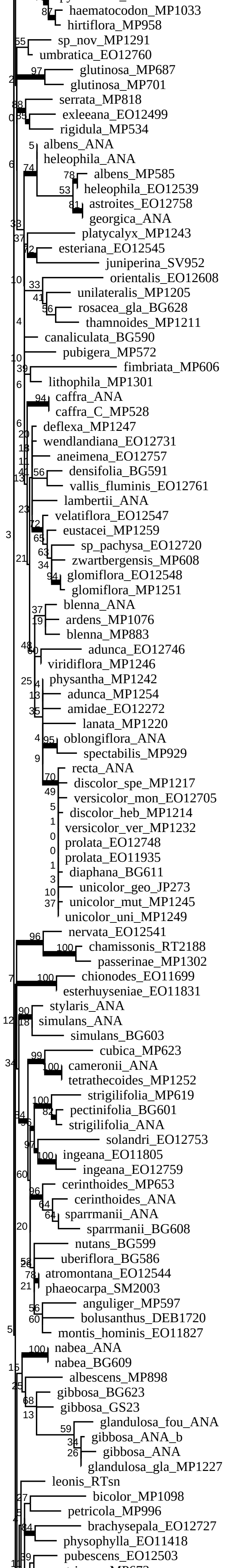
haematocodon_MP1033
hirtiflora_MP958
sp_nov_MP1291
umbratica_EO12760
glutinosa_MP687
glutinosa_MP701
serrata_MP818
exleeana_EO12499
rigidula_MP534
albens_ANA
heleophila_ANA
albens_MP585
heleophila_EO12539
astroites_EO12758
georgica_ANA
platycalyx_MP1243
esteriana_EO12545
juniperina_SV952
orientalis_EO12608
unilateralis_MP1205
rosacea_gla_BG628
thamnoides_MP1211
canaliculata_BG590
pubigera_MP572
fimbriata_MP606
lithophila_MP1301
caffra_ANA
caffra_C_MP528
deflexa_MP1247
wendlandiana_EO12731
aneimena_EO12757
densifolia_BG591
vallis_fluminis_EO12761
lambertii_ANA
velatiflora_EO12547
eustacei_MP1259
sp_pachysa_EO12720
zwartbergensis_MP608
glomiflora_EO12548
glomiflora_MP1251
blenna_ANA
ardens_MP1076
blenna_MP883
adunca_EO12746
viridiflora_MP1246
physantha_MP1242
adunca_MP1254
amidae_EO12272
lanata_MP1220
oblongiflora_ANA
spectabilis_MP929
recta_ANA
discolor_spe_MP1217
versicolor_mon_EO12705
discolor_heb_MP1214
versicolor_ver_MP1232
prolata_EO12748
prolata_EO11935
diaphana_BG611
unicolor_geo_JP273
unicolor_mut_MP1245
unicolor_uni_MP1249
nervata_EO12541
chamissonis_RT2188
passerinae_MP1302
chionodes_EO11699
esterhuyseniae_EO11831
stylaris_ANA
simulans_ANA
simulans_BG603
cubica_MP623
cameronii_ANA
tetrathecoides_MP1252
strigilifolia_MP619
pectinifolia_BG601
strigilifolia_ANA
solandri_EO12753
ingeana_EO11805
ingeana_EO12759
cerinthoides_MP653
cerinthoides_ANA
sparrmanii_ANA
sparrmanii_BG608
nutans_BG599
uberiflora_BG586
atromontana_EO12544
phaeocarpa_SM2003
anguliger_MP597
bolusanthus_DEB1720
montis_hominis_EO11827
nabea_ANA
nabea_BG609
albescens_MP898
gibbosa_BG623
gibbosa_GS23
glandulosa_fou_ANA
gibbosa_ANA_b
gibbosa_ANA
glandulosa_gla_MP1227
leonis_RTsn
bicolor_MP1098
petricola_MP996
brachysepala_EO12727
physophylla_EO11418
pubescens_EO12503

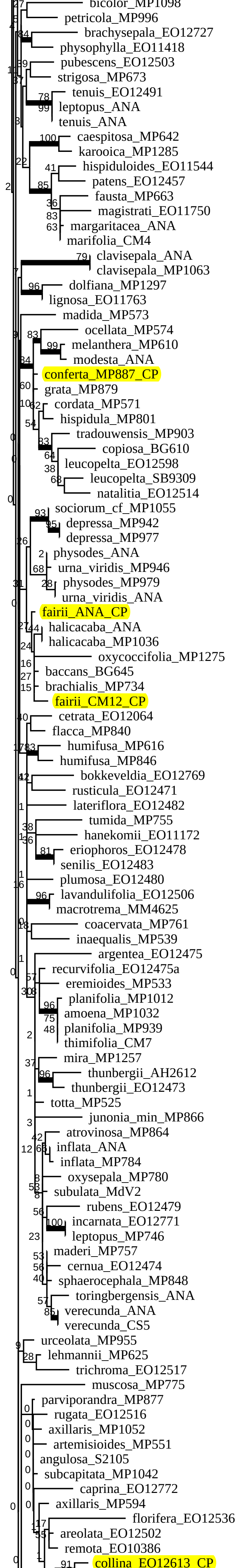

bicolor_MP1098
petricola_MP996
brachysepala_EO12727
physophylla_EO11418
pubescens_EO12503
strigosa_MP673
tenuis_EO12491
leptopus_ANA
tenuis_ANA
caespitosa_MP642
karooica_MP1285
hispiduloides_EO11544
patens_EO12457
fausta_MP663
magistrati_EO11750
margaritacea_ANA
marifolia_CM4
clavisepala_ANA
clavisepala_MP1063
dolfiana_MP1297
lignosa_EO11763
madida_MP573
ocellata_MP574
melanthera_MP610
modesta_ANA
conferta_MP887_CP
grata_MP879
cordata_MP571
hispidula_MP801
tradouwensis_MP903
copiosa_BG610
leucopelta_EO12598
leucopelta_SB9309
natalitia_EO12514
sociorum_cf_MP1055
depressa_MP942
depressa_MP977
physodes_ANA
urna_viridis_MP946
physodes_MP979
urna_viridis_ANA
fairii_ANA_CP
halicacaba_ANA
halicacaba_MP1036
oxycoccifolia_MP1275
baccans_BG645
brachialis_MP734
fairii_CM12_CP
cetrata_EO12064
flacca_MP840
humifusa_MP616
humifusa_MP846
bokkeveldia_EO12769
rusticula_EO12471
lateriflora_EO12482
tumida_MP755
hanekomii_EO11172
eriophoros_EO12478
senilis_EO12483
plumosa_EO12480
lavandulifolia_EO12506
macrotrema_MM4625
coacervata_MP761
inaequalis_MP539
argentea_EO12475
recurvifolia_EO12475a
eremioides_MP533
planifolia_MP1012
amoena_MP1032
planifolia_MP939
thimifolia_CM7
mira_MP1257
thunbergii_AH2612
thunbergii_EO12473
totta_MP525
junonia_min_MP866
atrovinosa_MP864
inflata_ANA
inflata_MP784
oxysepala_MP780
subulata_MdV2
rubens_EO12479
incarnata_EO12771
leptopus_MP746
maderi_MP757
cernua_EO12474
sphaerocephala_MP848
toringbergensis_ANA
verecunda_ANA
verecunda_CS5
urceolata_MP955
lehmannii_MP625
trichroma_EO12517
muscosa_MP775
parviporandra_MP877
rugata_EO12516
axillaris_MP1052
artemisioides_MP551
angulosa_S2105
subcapitata_MP1042
caprina_EO12772
axillaris_MP594
florifera_EO12536
areolata_EO12502
remota_EO10386
collina_EO12613_CP

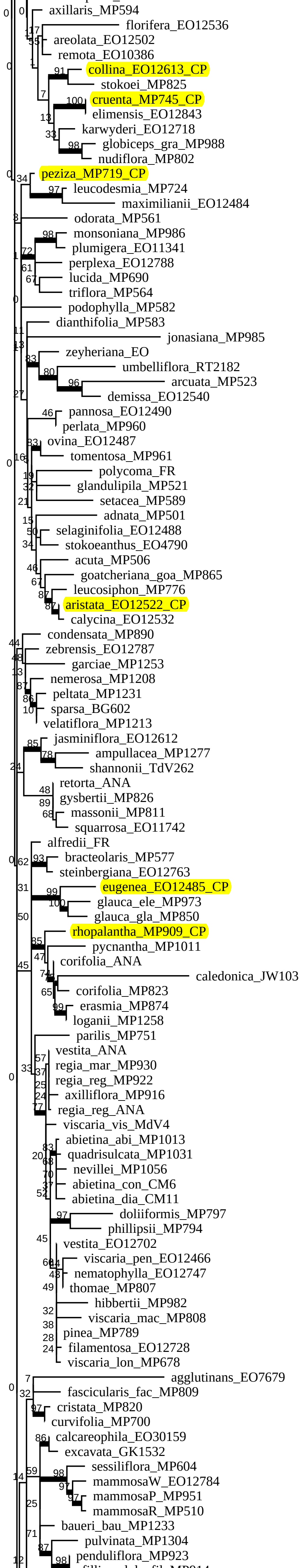
axillaris_MP594
florifera_EO12536
areolata_EO12502
remota_EO10386
collina_EO12613_CP
stokoei_MP825
cruenta_MP745_CP
elimensis_EO12843
karwyderi_EO12718
globiceps_gra_MP988
nudiflora_MP802
peziza_MP719_CP
leucodesmia_MP724
maximilianii_EO12484
odorata_MP561
monsoniana_MP986
plumigera_EO11341
perplexa_EO12788
lucida_MP690
triflora_MP564
podophylla_MP582
dianthifolia_MP583
jonasiana_MP985
zeyheriana_EO
umbelliflora_RT2182
arcuata_MP523
demissa_EO12540
pannosa_EO12490
perlata_MP960
ovina_EO12487
tomentosa_MP961
polycoma_FR
glandulipila_MP521
setacea_MP589
adnata_MP501
selaginifolia_EO12488
stokoeanthus_EO4790
acuta_MP506
goatcheriana_goa_MP865
leucosiphon_MP776
aristata_EO12522_CP
calycina_EO12532
condensata_MP890
zebrensis_EO12787
garciae_MP1253
nemerosa_MP1208
peltata_MP1231
sparsa_BG602
velatiflora_MP1213
jasminiflora_EO12612
ampullacea_MP1277
shannonii_TdV262
retorta_ANA
gysbertii_MP826
massonii_MP811
squarrosa_EO11742
alfredii_FR
bracteolaris_MP577
steinbergiana_EO12763
eugenea_EO12485_CP
glauca_ele_MP973
glauca_gla_MP850
rhopalantha_MP909_CP
pycnantha_MP1011
corifolia_ANA
caledonica_JW103
corifolia_MP823
erasmia_MP874
loganii_MP1258
parilis_MP751
vestita_ANA
regia_mar_MP930
regia_reg_MP922
axilliflora_MP916
regia_reg_ANA
viscaria_vis_MdV4
abietina_abi_MP1013
quadrisulcata_MP1031
nevillei_MP1056
abietina_con_CM6
abietina_dia_CM11
doliiformis_MP797
phillipsii_MP794
vestita_EO12702
viscaria_pen_EO12466
nematophylla_EO12747
thomae_MP807
hibbertii_MP982
viscaria_mac_MP808
pinea_MP789
filamentosa_EO12728
viscaria_lon_MP678
agglutinans_EO7679
fascicularis_fac_MP809
cristata_MP820
curvifolia_MP700
calcareophila_EO30159
excavata_GK1532
sessiliflora_MP604
mammosaW_EO12784
mammosaP_MP951
mammosaR_MP510
baueri_bau_MP1233
pulvinata_MP1304
penduliflora_MP923

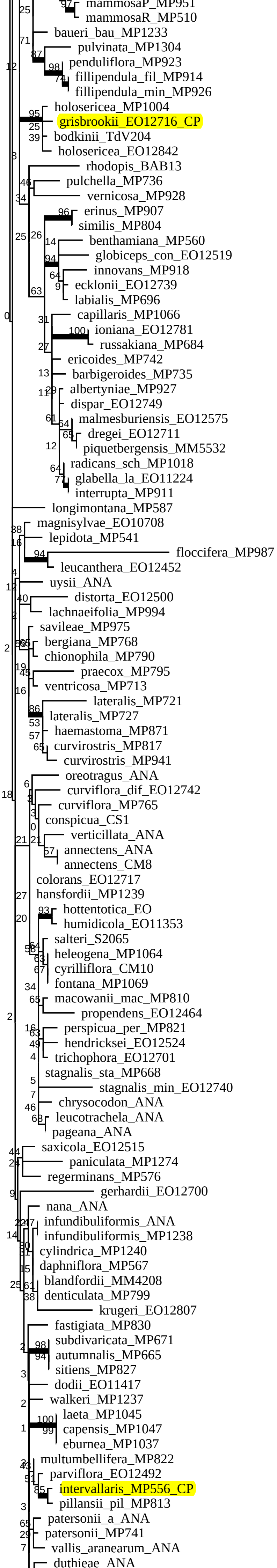
mammosaP_MP951
mammosaR_MP510
baueri_bau_MP1233
pulvinata_MP1304
penduliflora_MP923
fillipendula_fil_MP914
fillipendula_min_MP926
holosericea_MP1004
grisbrookii_EO12716_CP
bodkinii_TdV204
holosericea_EO12842
rhodopis_BAB13
pulchella_MP736
vernicosa_MP928
erinus_MP907
similis_MP804
benthamiana_MP560
globiceps_con_EO12519
innovans_MP918
ecklonii_EO12739
labialis_MP696
capillaris_MP1066
ioniana_EO12781
russakiana_MP684
ericoides_MP742
barbigeroides_MP735
albertyniae_MP927
dispar_EO12749
malmesburiensis_EO12575
dregei_EO12711
piquetbergensis_MM5532
radicans_sch_MP1018
glabella_la_EO11224
interrupta_MP911
longimontana_MP587
magnisylvae_EO10708
lepidota_MP541
floccifera_MP987
leucanthera_EO12452
uysii_ANA
distorta_EO12500
lachnaeifolia_MP994
savileae_MP975
bergiana_MP768
chionophila_MP790
praecox_MP795
ventricosa_MP713
lateralis_MP721
lateralis_MP727
haemastoma_MP871
curvirostris_MP817
curvirostris_MP941
oreotragus_ANA
curviflora_dif_EO12742
curviflora_MP765
conspicua_CS1
verticillata_ANA
annectens_ANA
annectens_CM8
colorans_EO12717
hansfordii_MP1239
hottentotica_EO
humidicola_EO11353
salteri_S2065
heleogena_MP1064
cyrilliflora_CM10
fontana_MP1069
macowanii_mac_MP810
propendens_EO12464
perspicua_per_MP821
hendricksei_EO12524
trichophora_EO12701
stagnalis_sta_MP668
stagnalis_min_EO12740
chrysocodon_ANA
leucotrachela_ANA
pageana_ANA
saxicola_EO12515
paniculata_MP1274
regerminans_MP576
gerhardii_EO12700
nana_ANA
infundibuliformis_ANA
infundibuliformis_MP1238
cylindrica_MP1240
daphniflora_MP567
blandfordii_MM4208
denticulata_MP799
krugeri_EO12807
fastigiata_MP830
subdivaricata_MP671
autumnalis_MP665
sitiens_MP827
dodii_EO11417
walkeri_MP1237
laeta_MP1045
capensis_MP1047
eburnea_MP1037
multumbellifera_MP822
parviflora_EO12492
intervallaris_MP556_CP
pillansii_pil_MP813
patersonii_a_ANA
patersonii_MP741
vallis_aranearum_ANA
duthieae_ANA

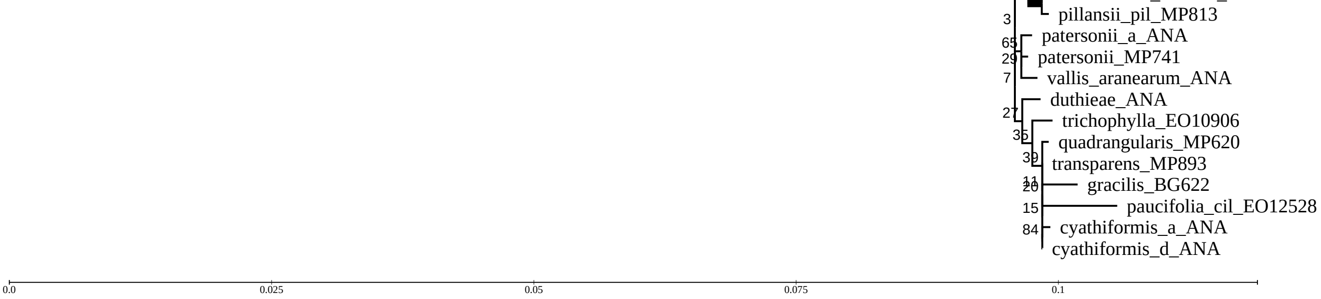

pillansii_pil_MP813
patersonii_a_ANA
patersonii_MP741
vallis_aranearum_ANA
duthieae_ANA
trichophylla_EO10906
quadrangularis_MP620
transparens_MP893
gracilis_BG622
paucifolia_cil_EO12528
cyathiformis_a_ANA
cyathiformis_d_ANA
0.0
0.025
0.05
0.075
0.1
