# Supplementary figures and images for: The biodiversity hotspot as evolutionary hot-bed: spectacular radiation of Erica in the Cape Floristic Region
Source: BMC Evol Biol. 2016 Sep 17;16:190. doi: 10.1186/s12862-016-0764-3 (PMC5027107; doi:10.1186/s12862-016-0764-3)

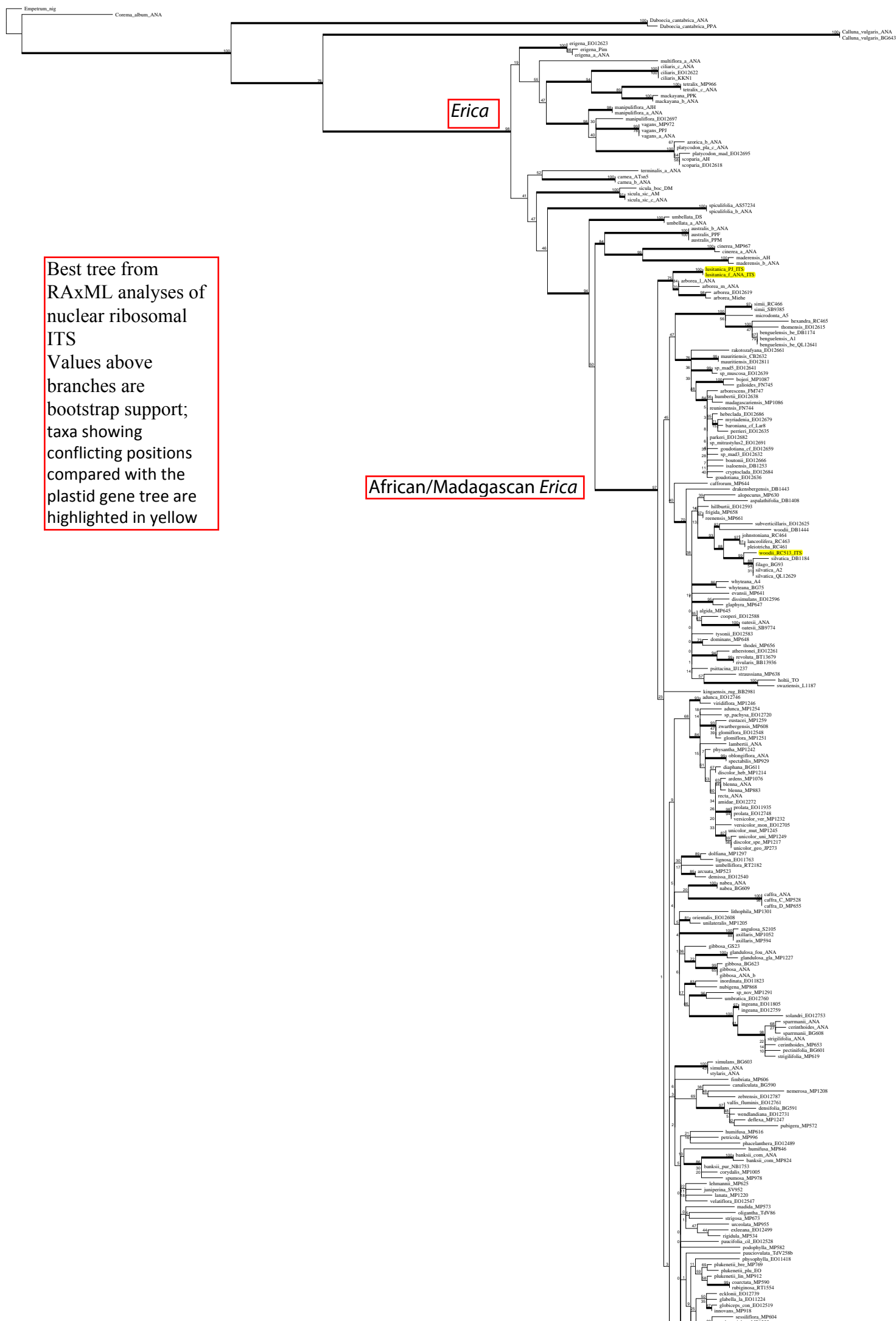

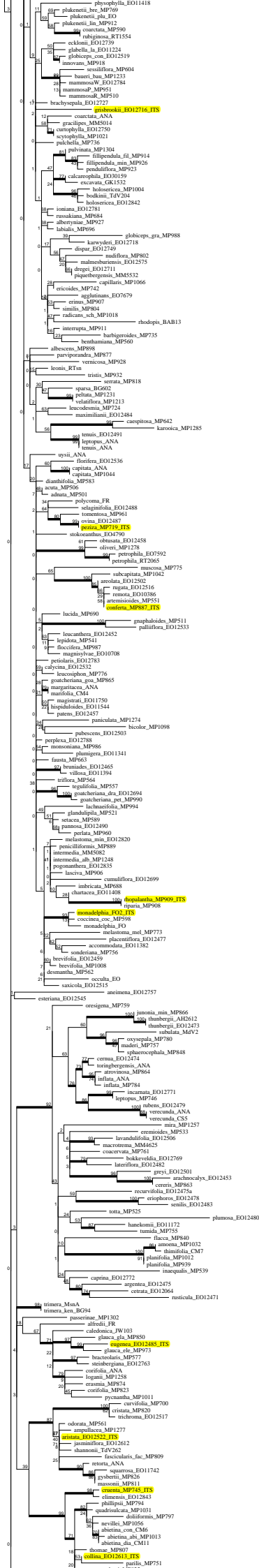

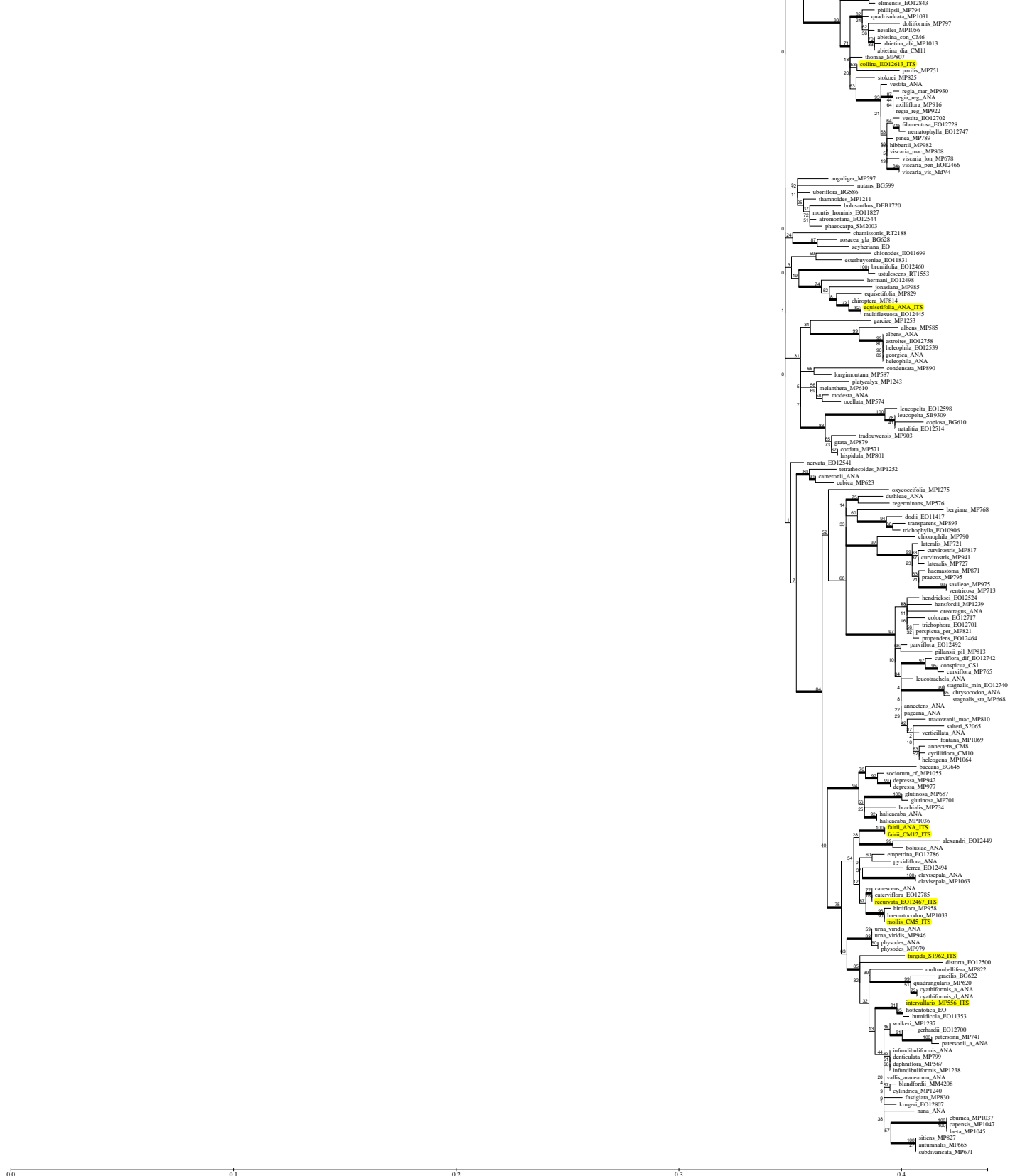

Supplement: Additional file 2: Figure S1. — Phylogenetic hypotheses: best trees with bootstrap support values from RAxML analyses of a) concatenated plastid data and b) from nuclear ribosomal ITS (with taxa showing conflicting positions according to the two gene trees highlighted in yellow); and c) and d) of the combined data (excluding conflicting taxa): c) with and d) without Erica pauciovulata (exclusion of which leads to increased support for the Cape clade from 70 % to 89 %). (ZIP 8409 kb) [file 12862_2016_764_MOESM2_ESM.zip › add 4/S1b_Figure_RAxML_ITS_new.pdf]

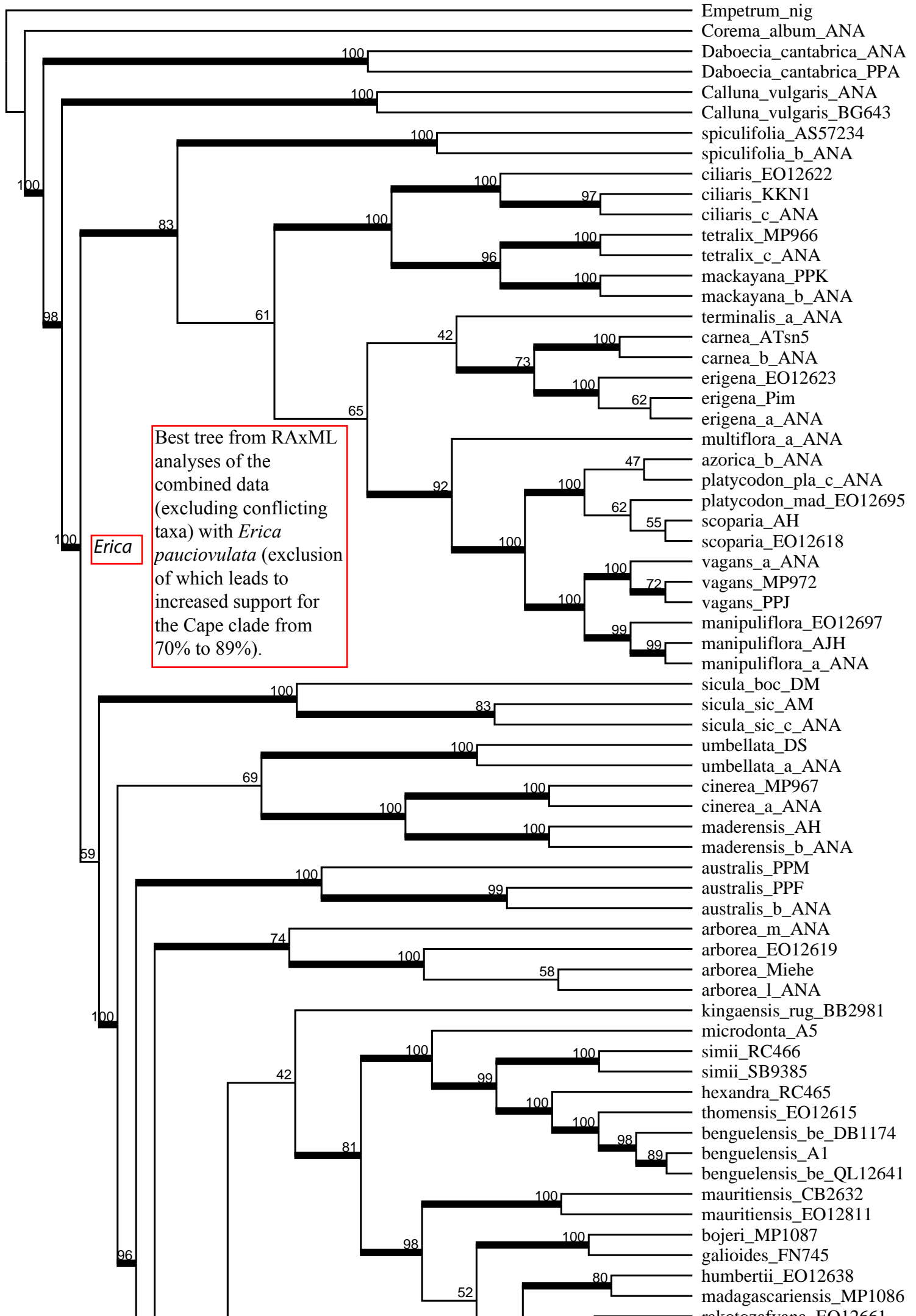

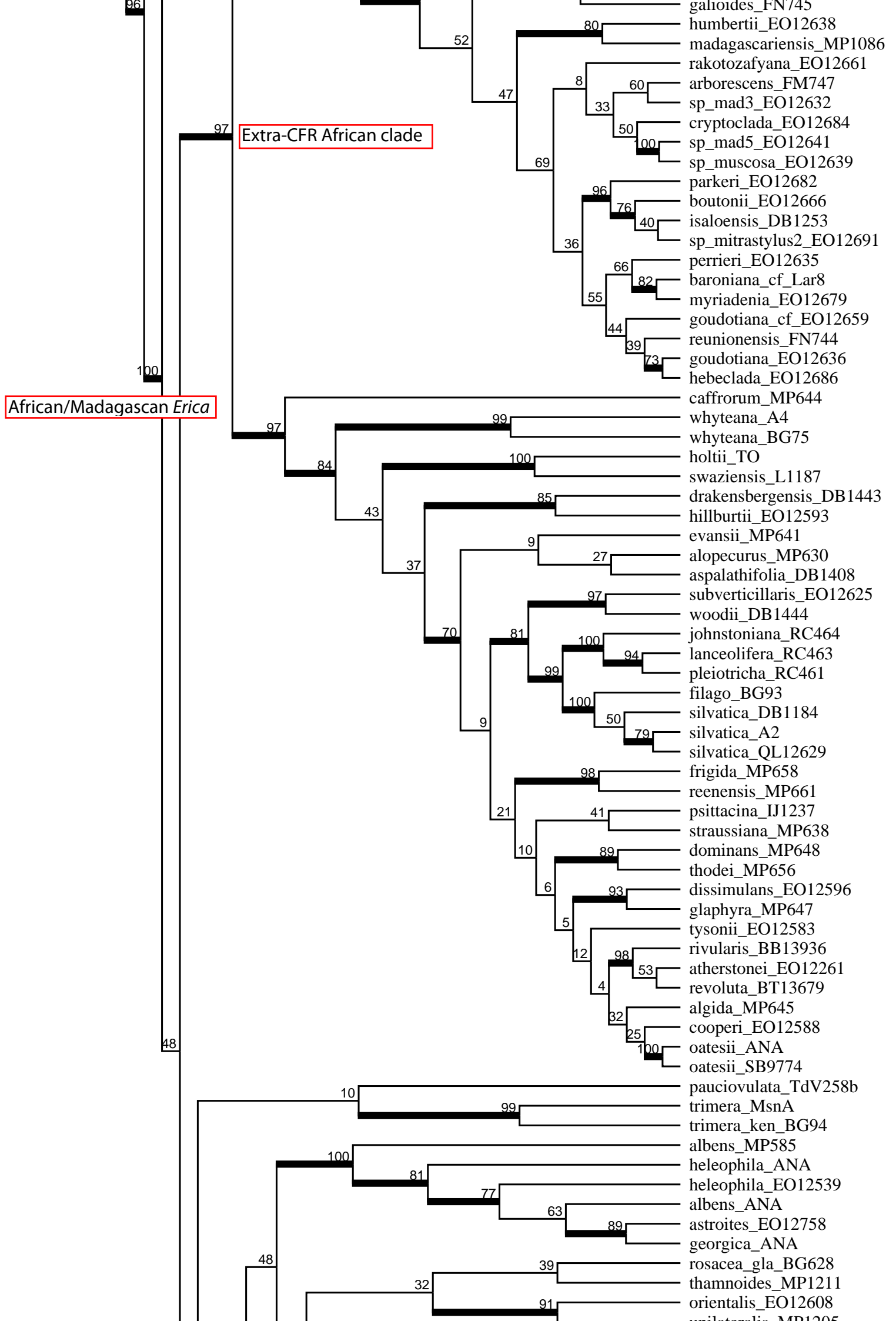

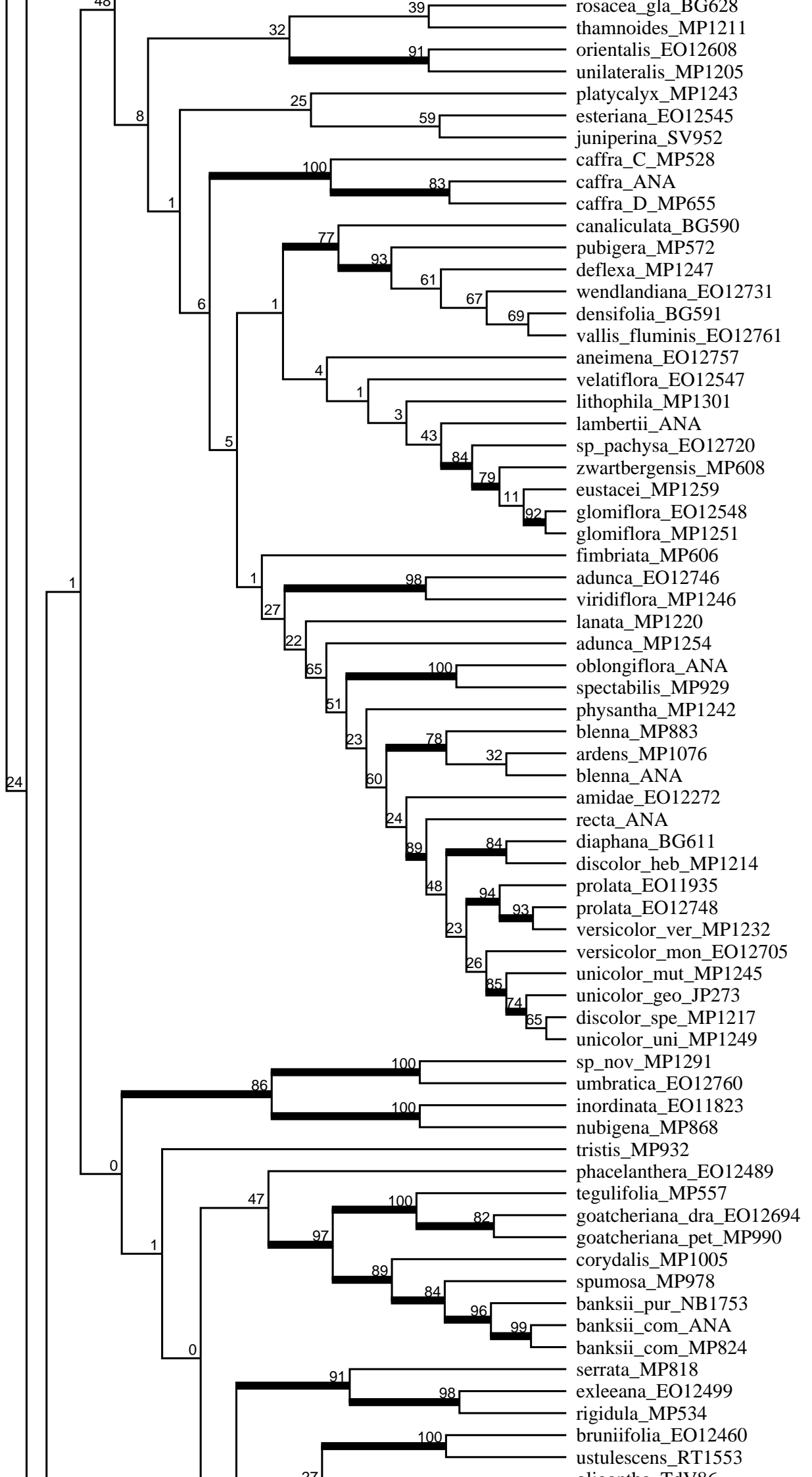

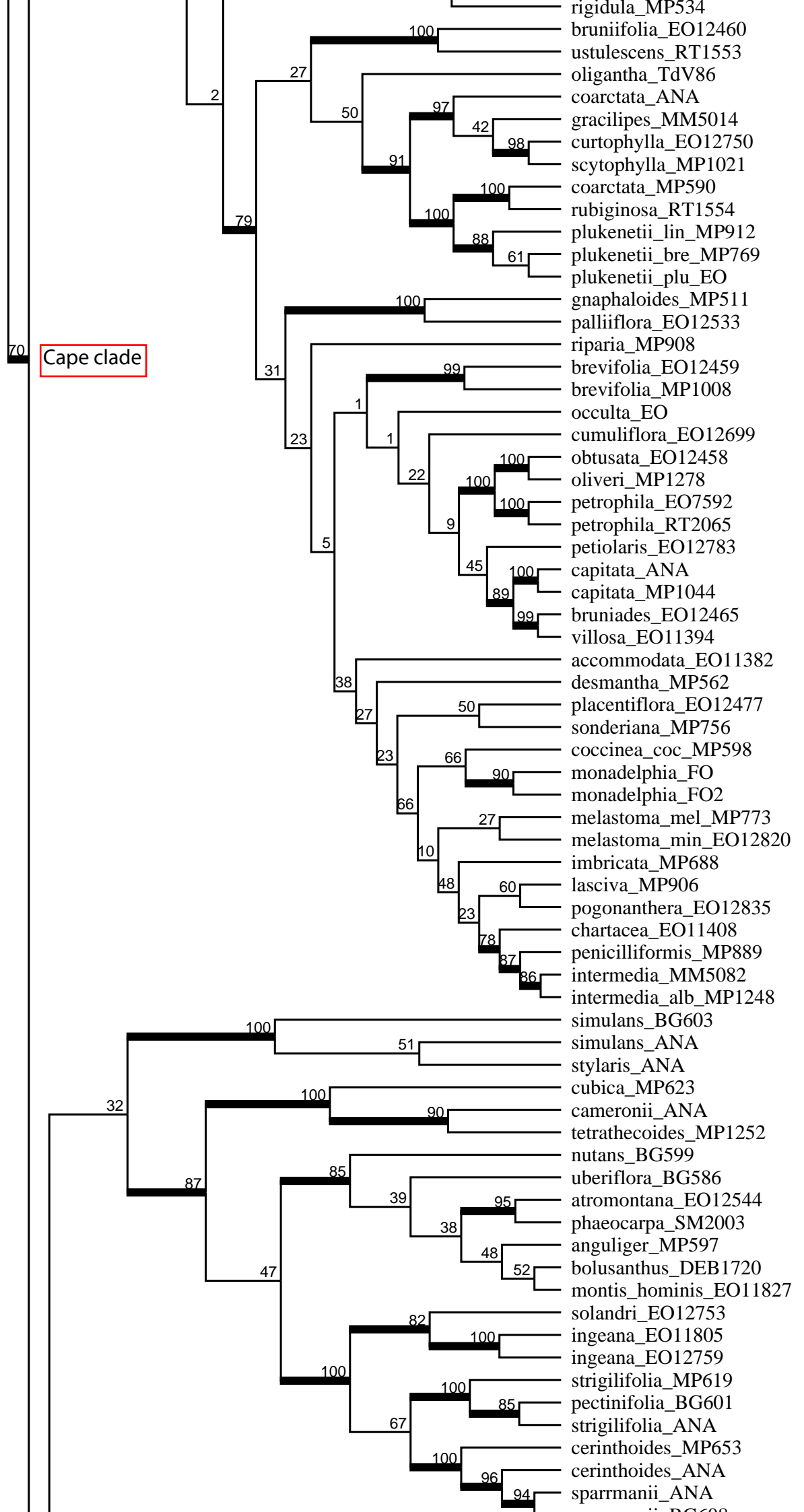

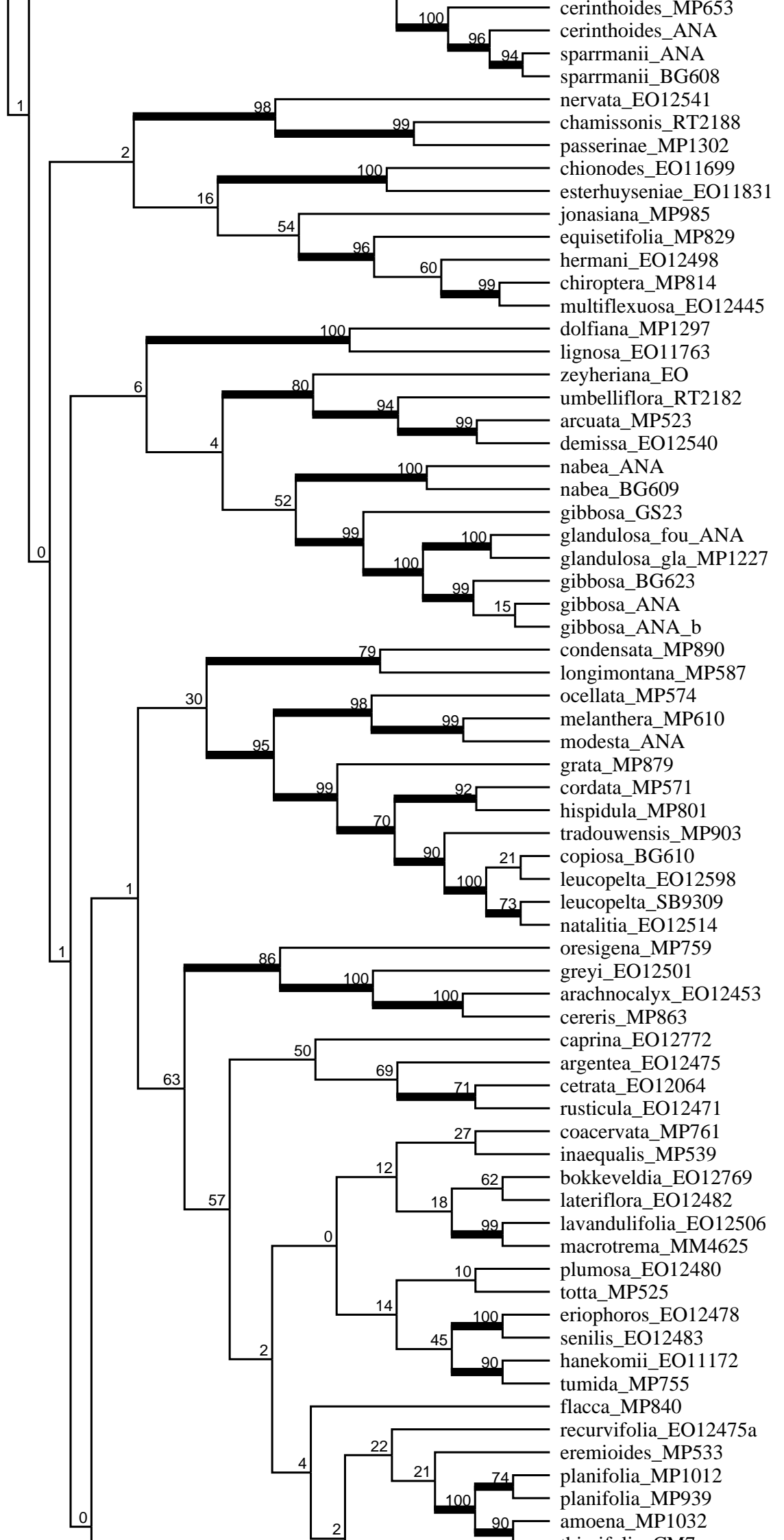

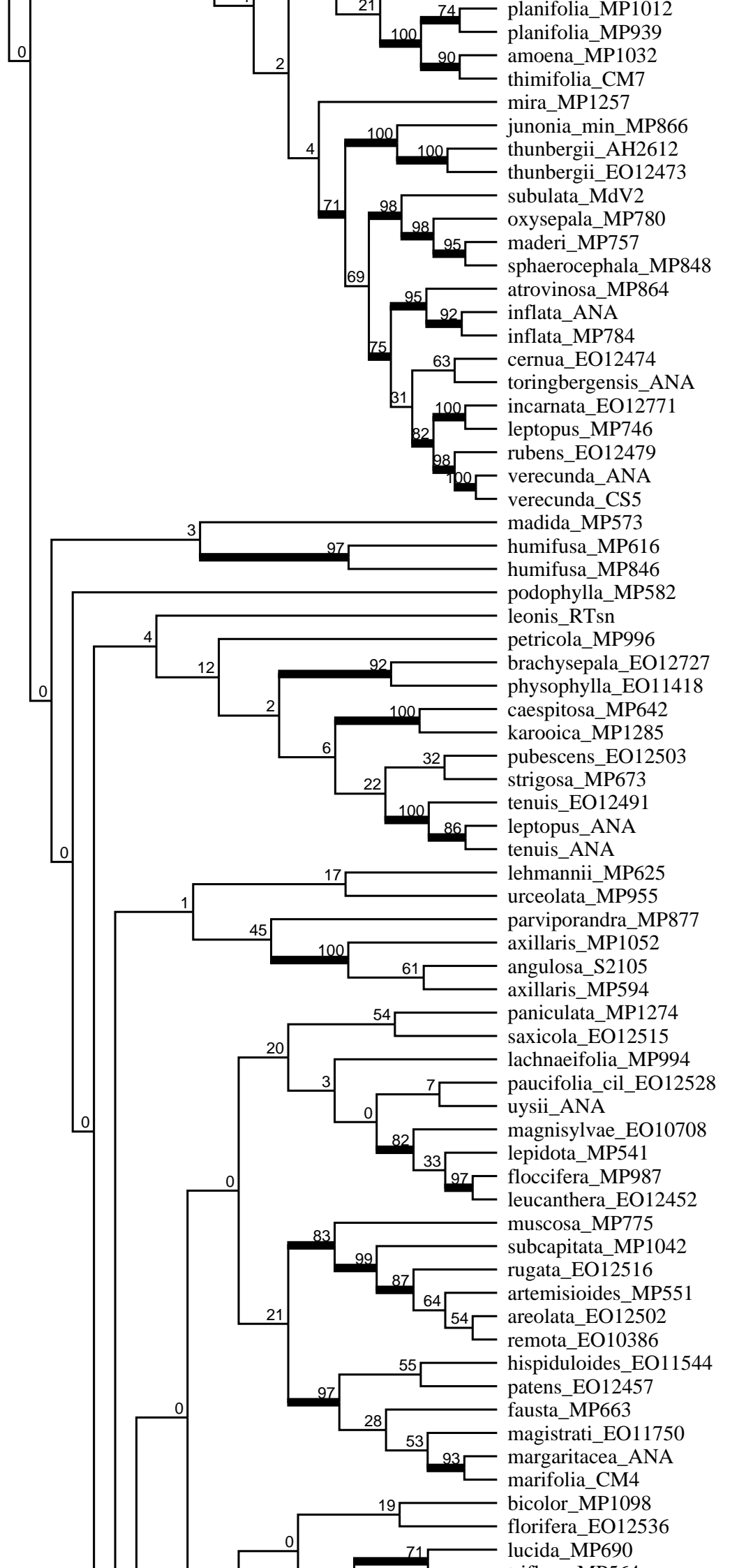

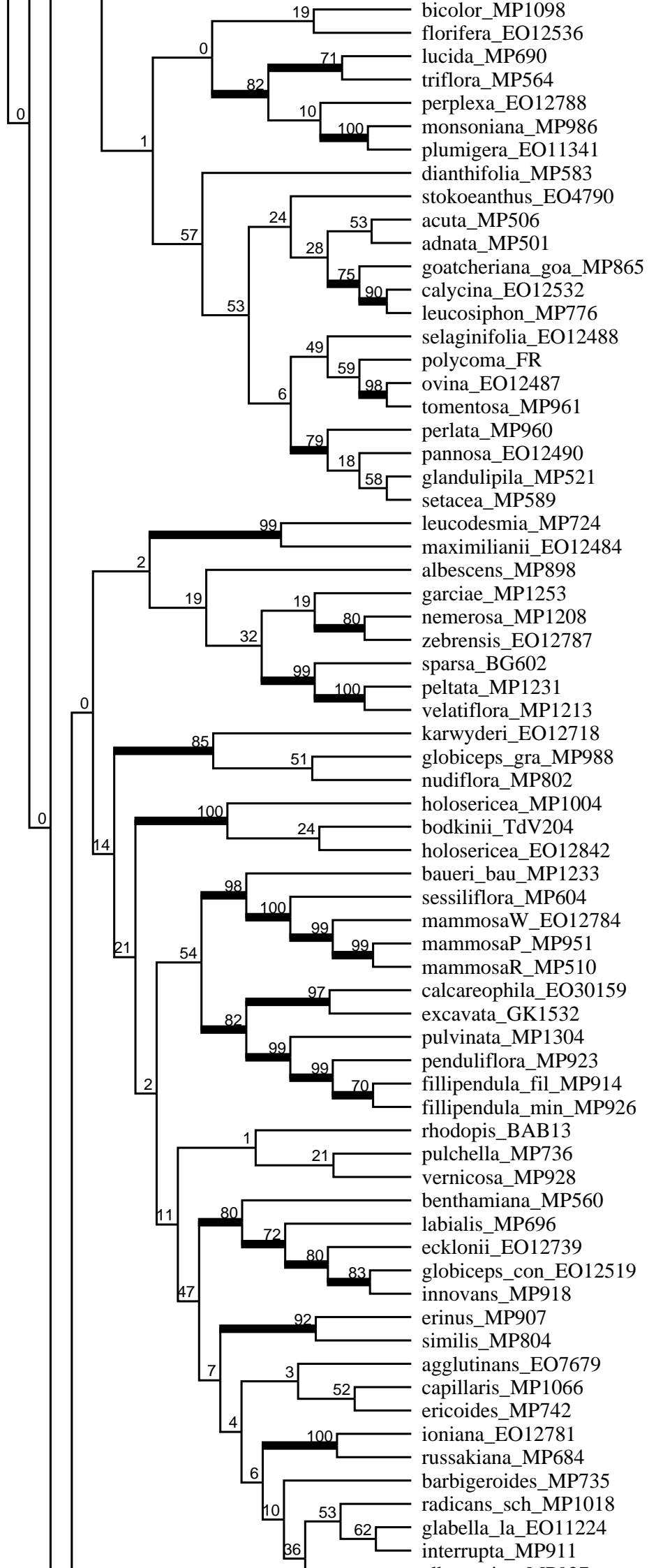

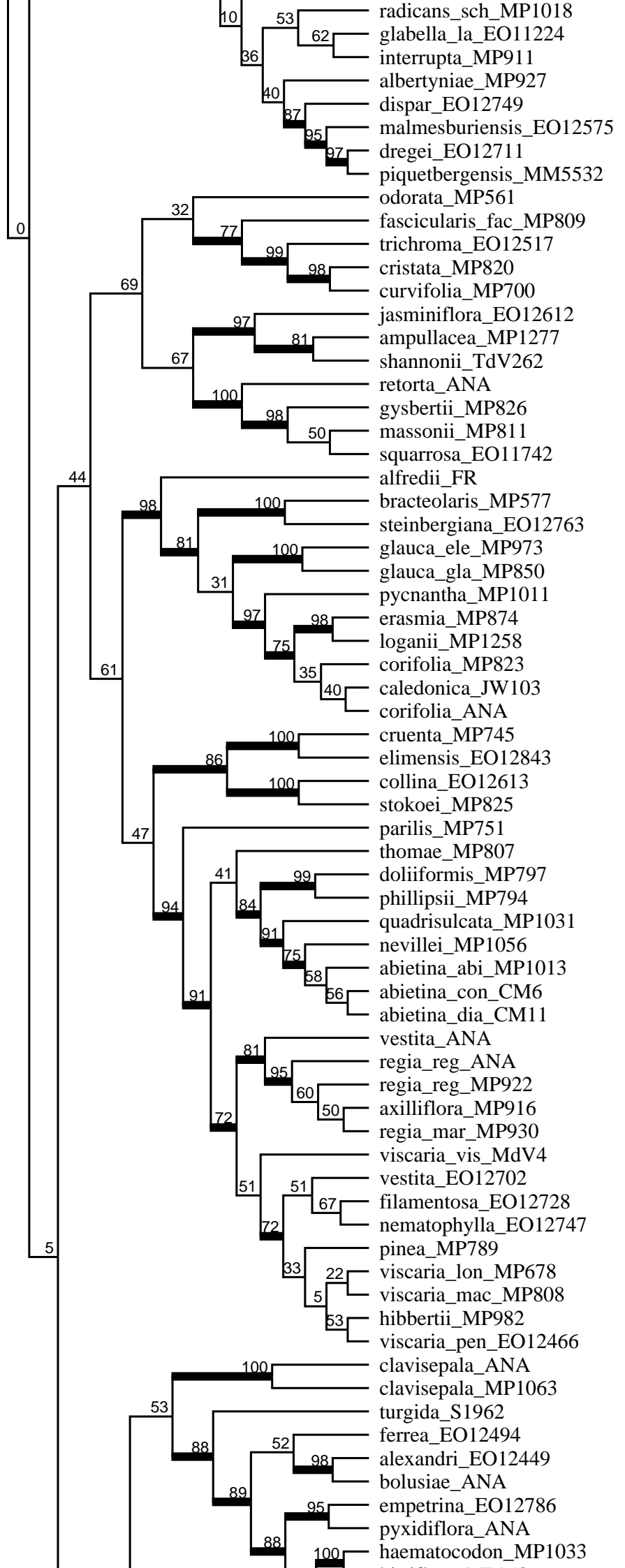

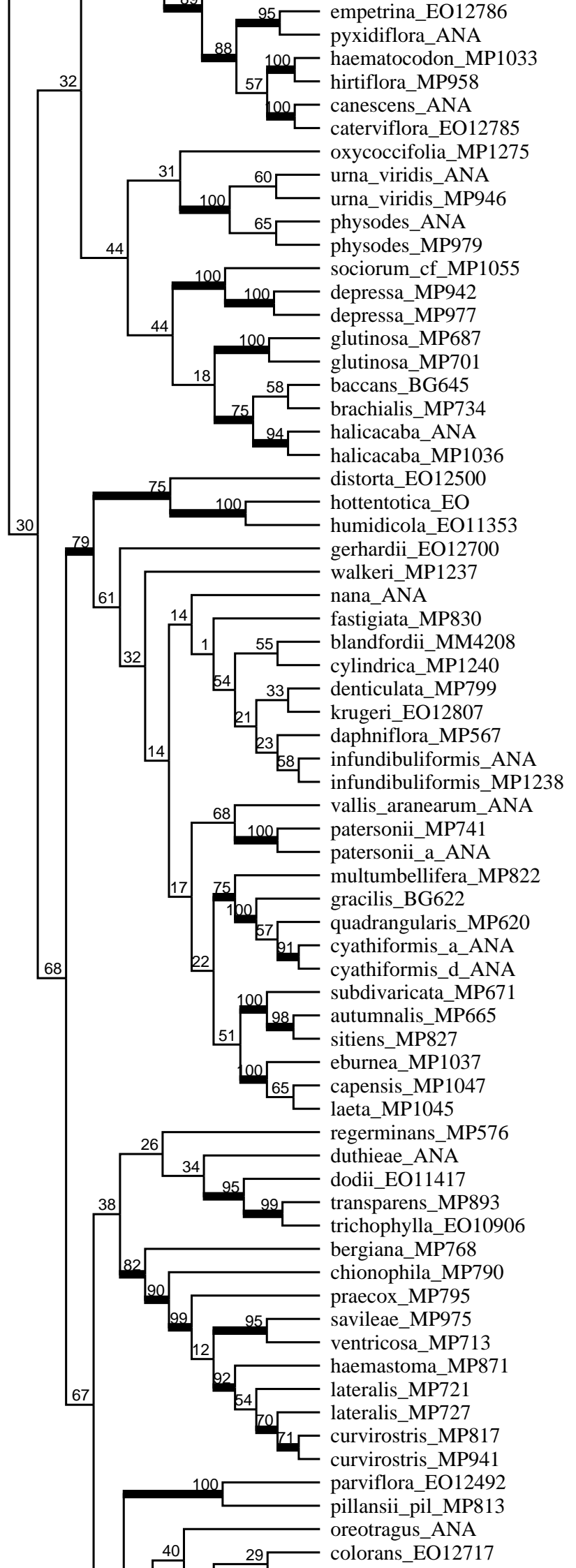

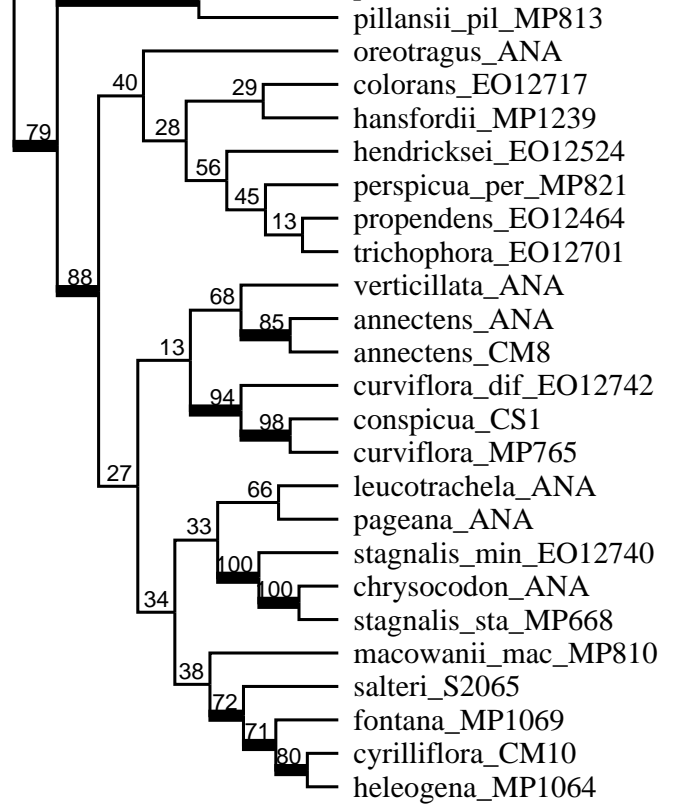

Supplement: Additional file 2: Figure S1. — Phylogenetic hypotheses: best trees with bootstrap support values from RAxML analyses of a) concatenated plastid data and b) from nuclear ribosomal ITS (with taxa showing conflicting positions according to the two gene trees highlighted in yellow); and c) and d) of the combined data (excluding conflicting taxa): c) with and d) without Erica pauciovulata (exclusion of which leads to increased support for the Cape clade from 70 % to 89 %). (ZIP 8409 kb) [file 12862_2016_764_MOESM2_ESM.zip › add 4/S1c_Figure_RAxML_Combined_new.pdf]

Figure S2d

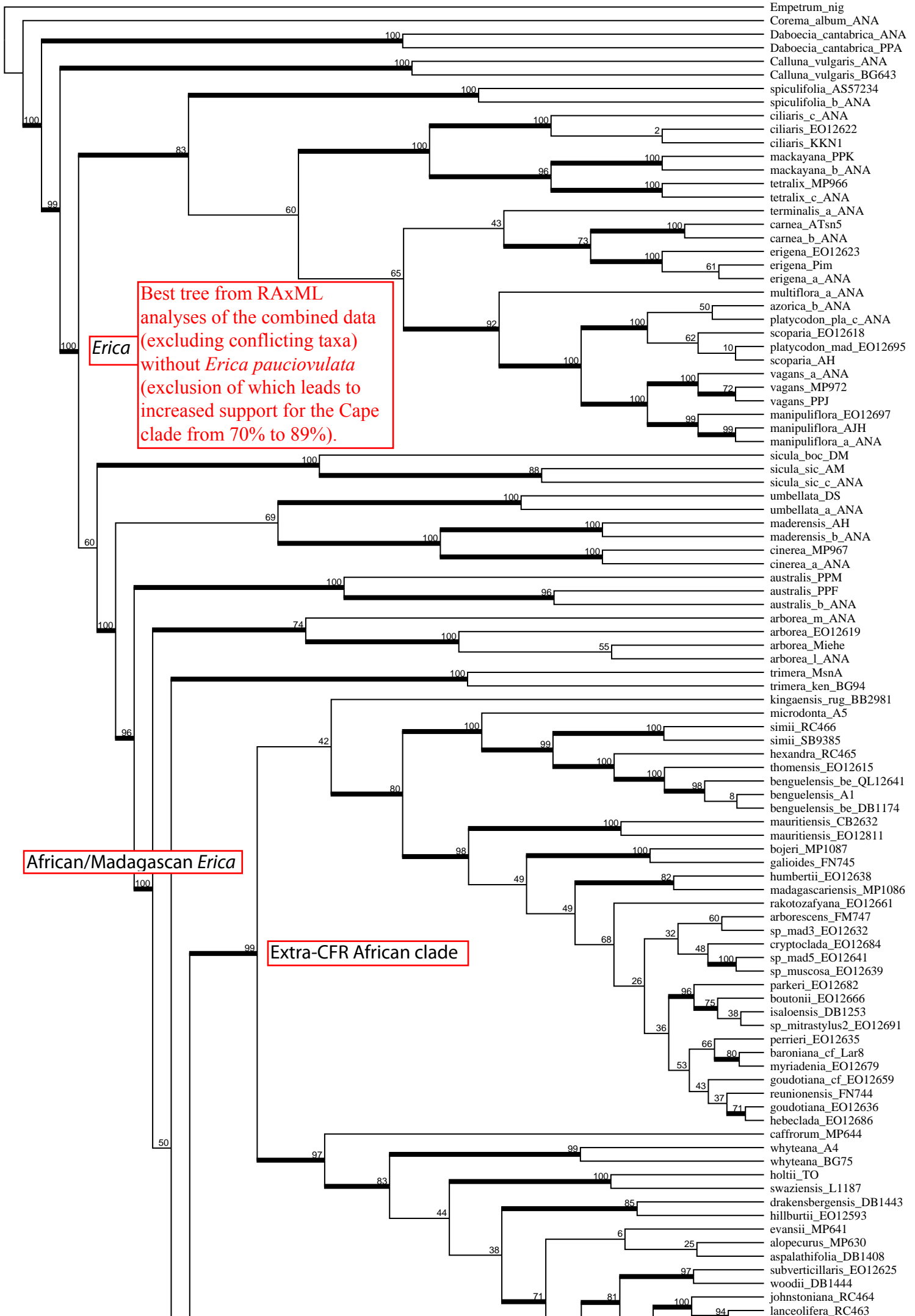

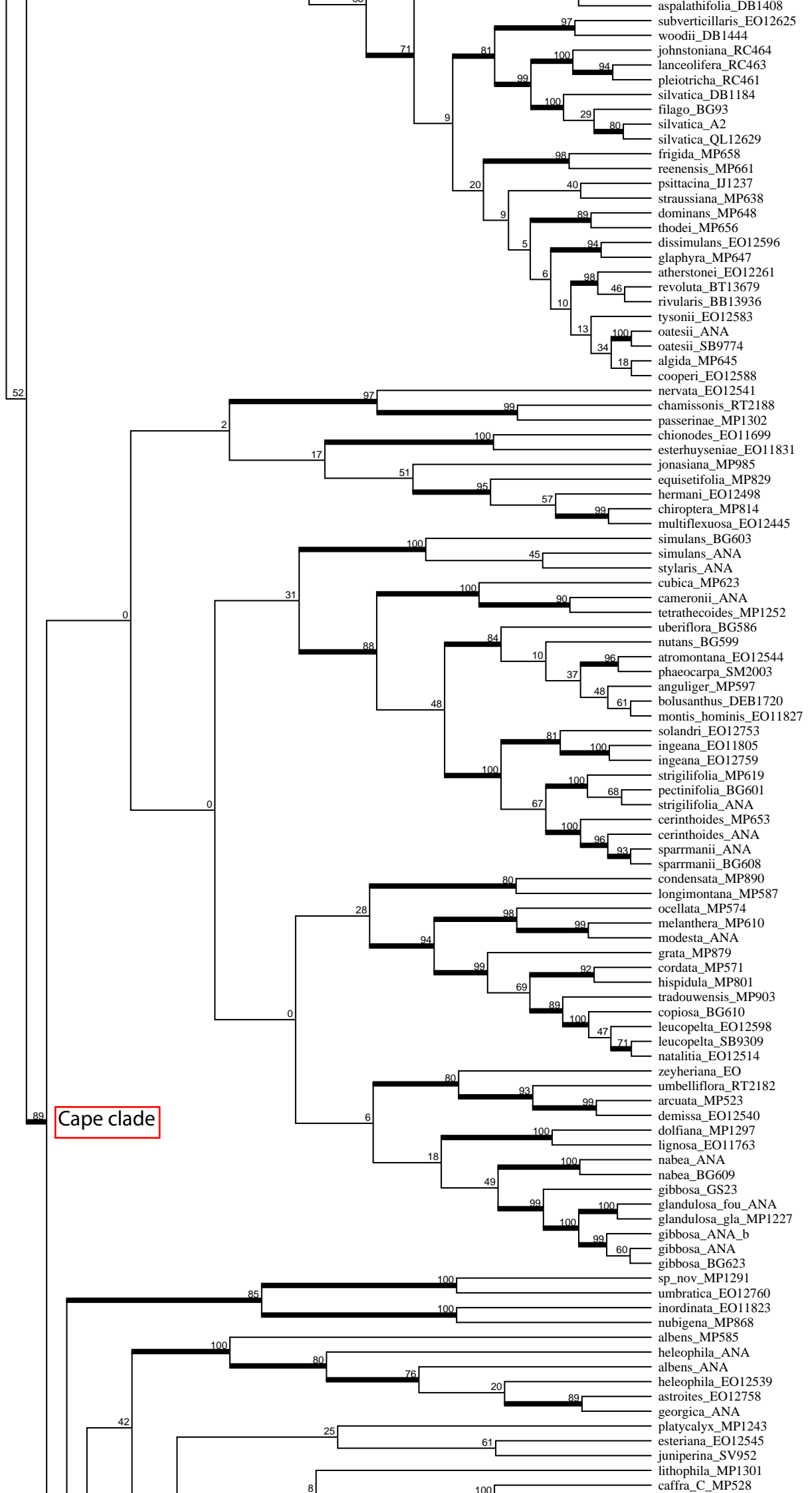

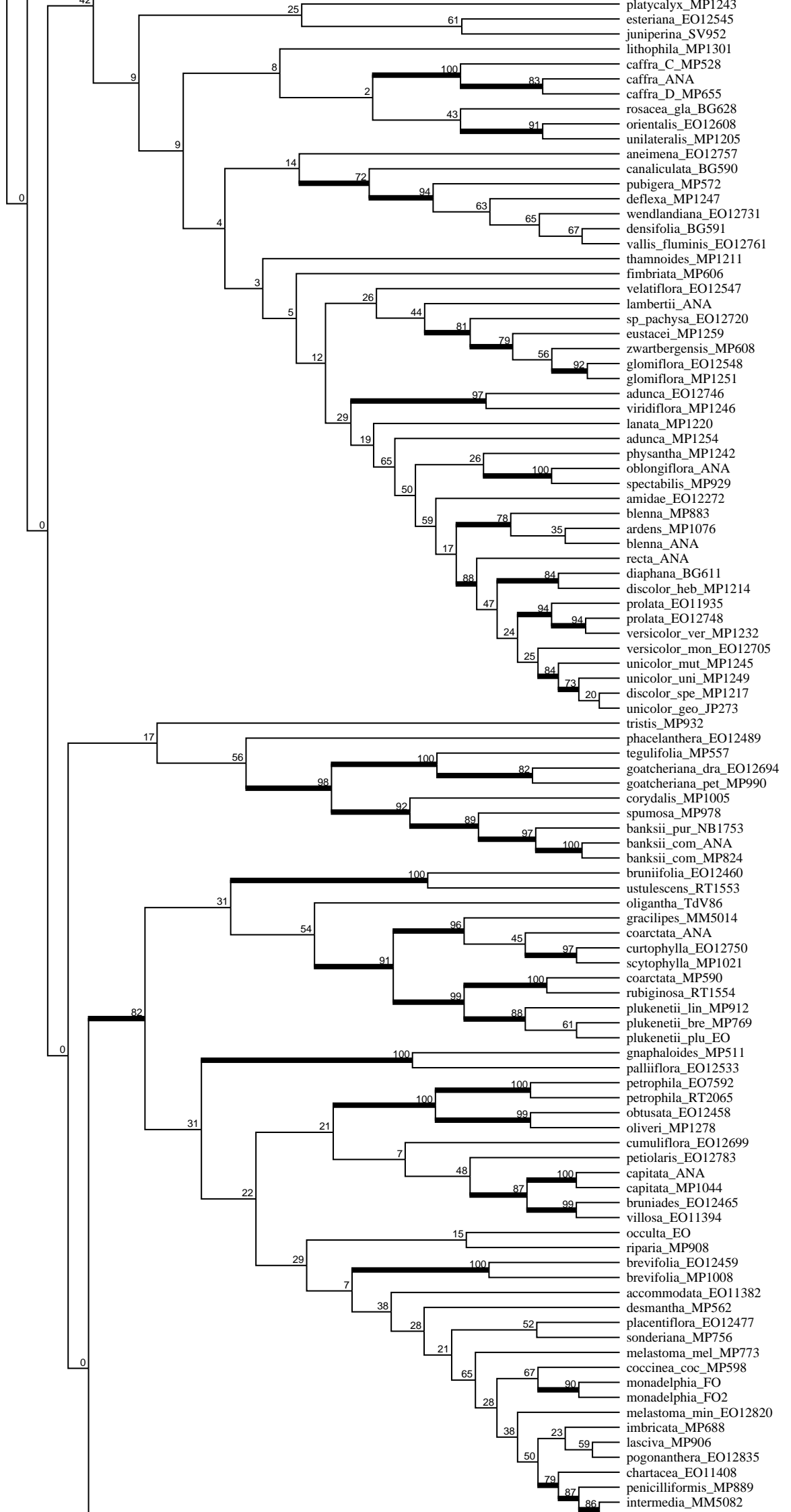

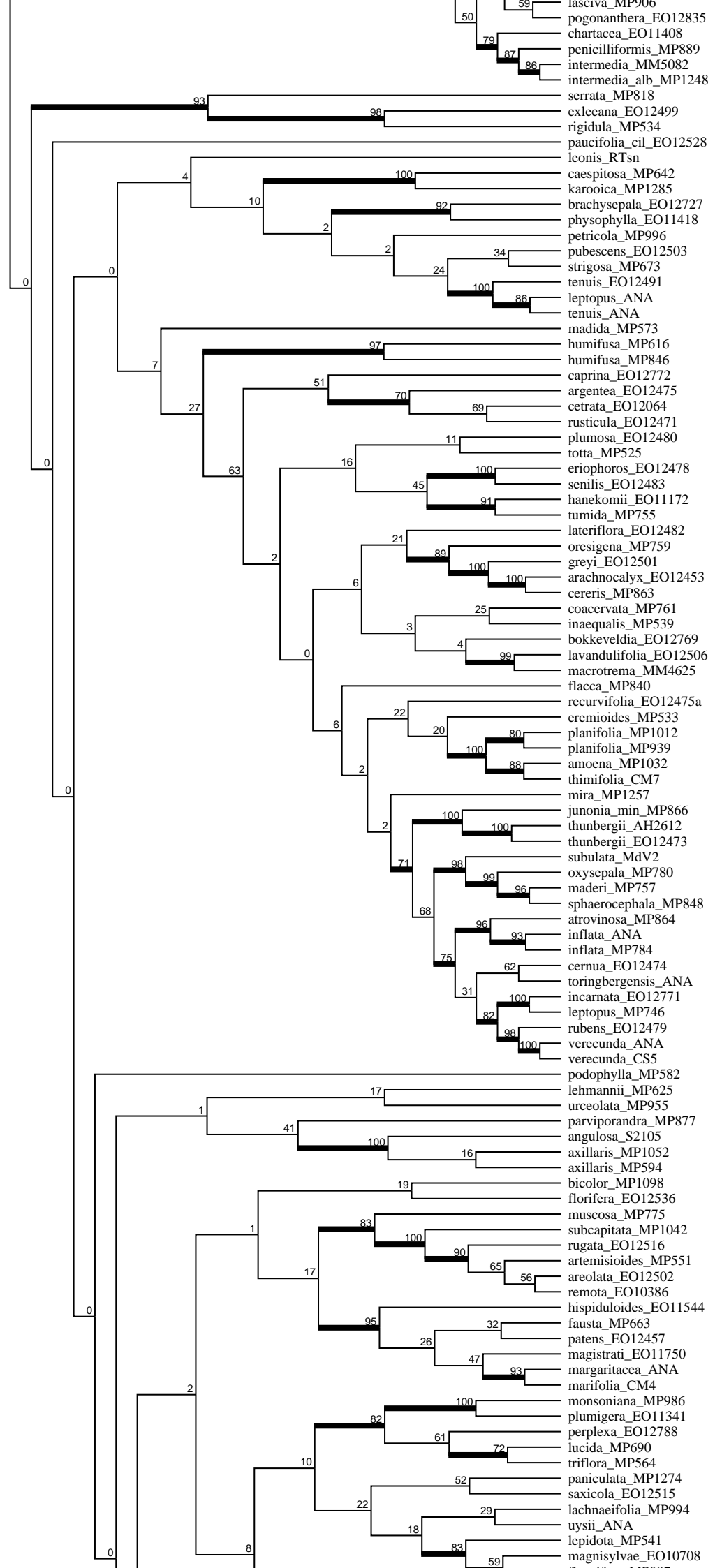

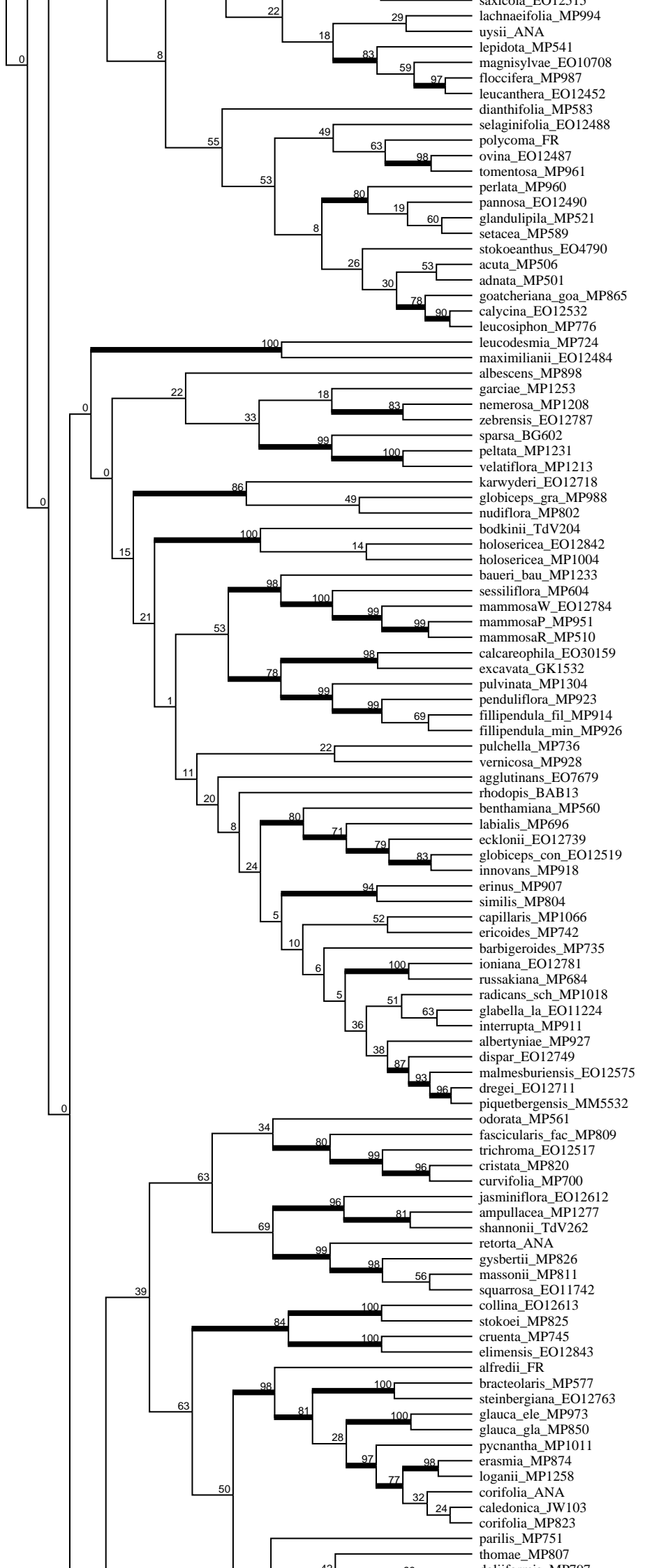

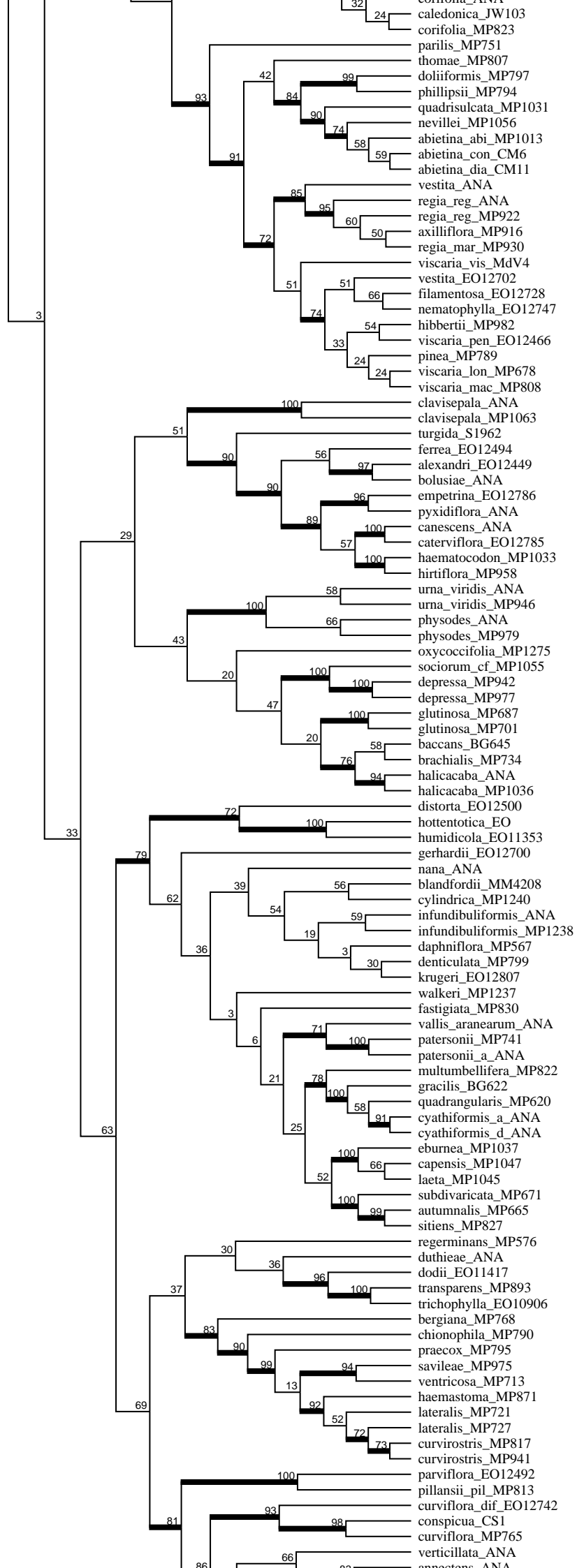

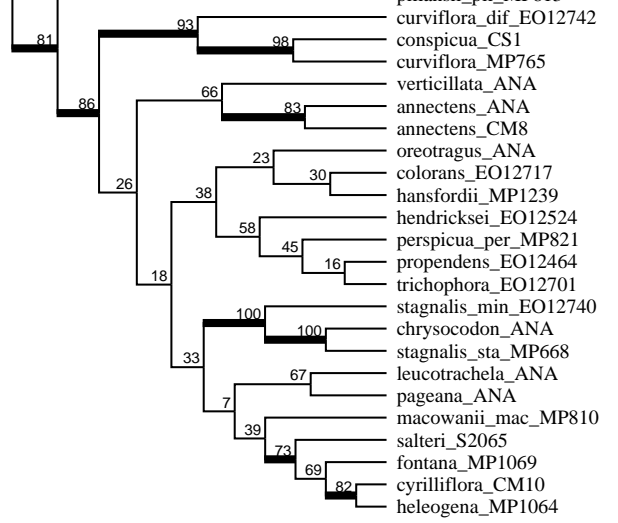

Supplement: Additional file 2: Figure S1. — Phylogenetic hypotheses: best trees with bootstrap support values from RAxML analyses of a) concatenated plastid data and b) from nuclear ribosomal ITS (with taxa showing conflicting positions according to the two gene trees highlighted in yellow); and c) and d) of the combined data (excluding conflicting taxa): c) with and d) without Erica pauciovulata (exclusion of which leads to increased support for the Cape clade from 70 % to 89 %). (ZIP 8409 kb) [file 12862_2016_764_MOESM2_ESM.zip › add 4/S1d_Figure_RAxML_Combined_no_pauci_new.pdf]

Figure S3b: Probabilities of overall numbers of diversification shifts inferred using BAMM.

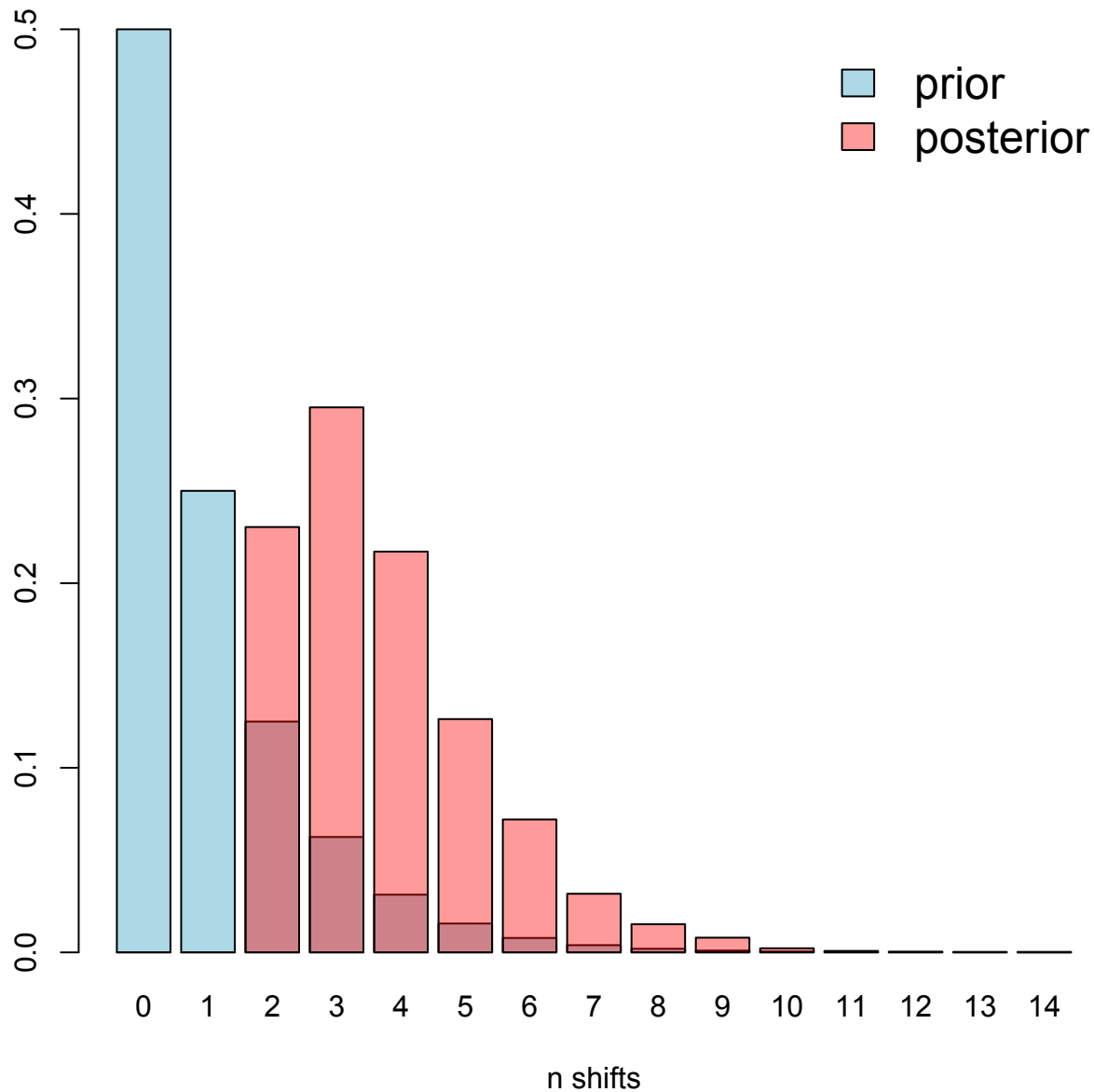

Supplement: Additional file 5: Figures S3. — BAMM diversification rate results: a) BAMM tree as presented in Fig. 1, including labels for tips and nodes referred to in the text; branches subtending Erica, Calluna and Daboecia are not to scale. b) Probabilities of overall numbers of diversification shifts inferred using BAMM. (ZIP 2444 kb) [file 12862_2016_764_MOESM5_ESM.zip › add 6/S3b_Figure_BAMM diversification shifts probabilities_2016.pdf]
