# Supplementary material for: The biodiversity hotspot as evolutionary hot-bed: spectacular radiation of Erica in the Cape Floristic Region
Source: BMC Evol Biol. 2016 Sep 17;16:190. doi: 10.1186/s12862-016-0764-3 (PMC5027107; doi:10.1186/s12862-016-0764-3)
Supplement: Additional file 3: Figure S2. — Relaxed clock molecular dating results: a) age estimates for clades within Erica inferred using RELTIME [24] with the best tree from RAxML (Additional file 2: Figure S1c); b) and c) phylogeny and relaxed clock molecular dating age estimates for clades within Erica inferred using BEAST [23] on a reduced matrix of 62 taxa b) with and c) without additional constraint based on microfossil evidence (error bars represent 95 % Posterior Probability (PP) intervals; PP clade support is indicated at nodes). (ZIP 11562 kb) [file 12862_2016_764_MOESM3_ESM.zip › add 5/S2c_Figure_BEAST_root_new.pdf]

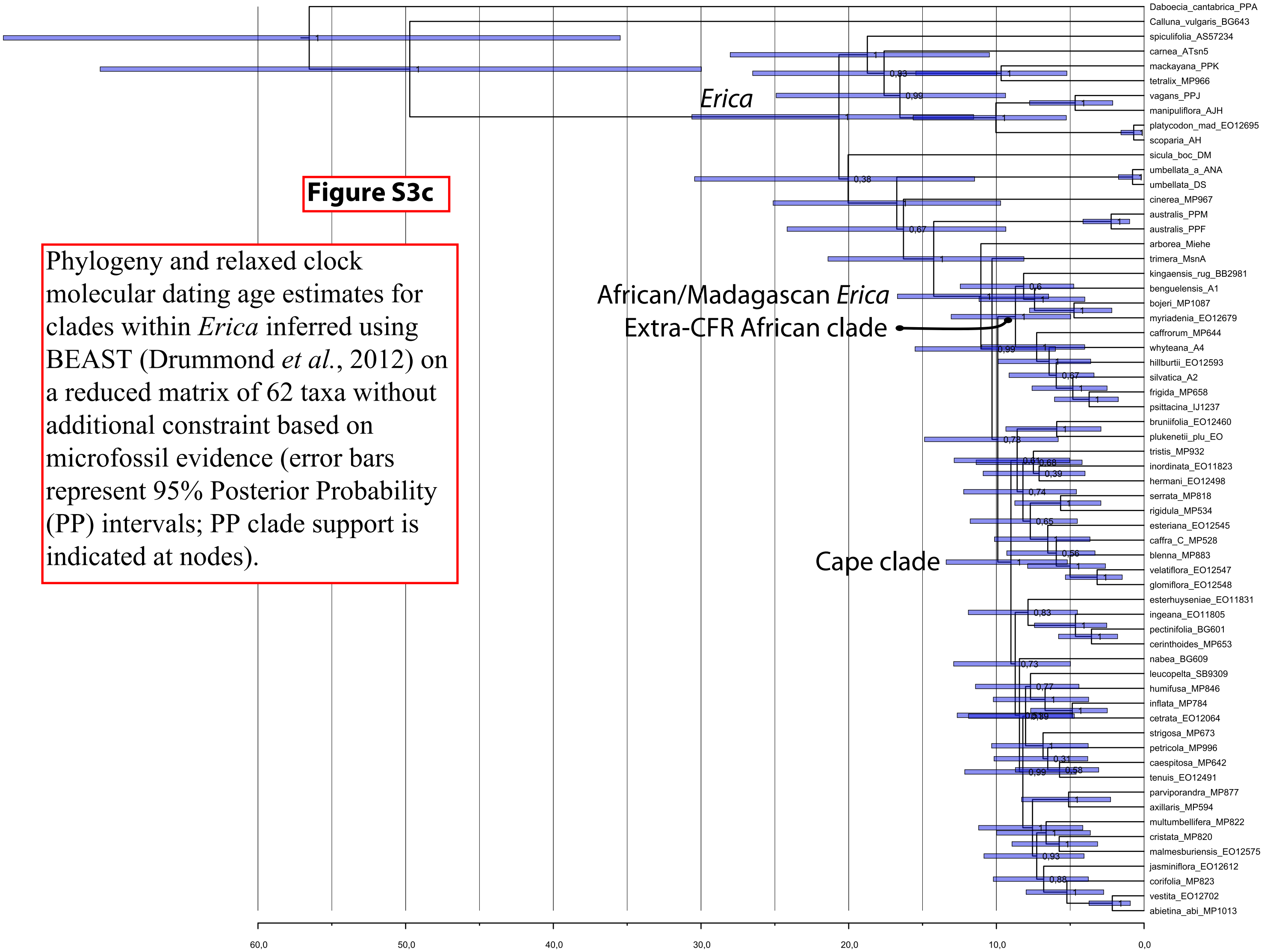

Figure S3c

Phylogeny and relaxed clock molecular dating age estimates for clades within *Erica* inferred using BEAST (Drummond *et al.*, 2012) on a reduced matrix of 62 taxa without additional constraint based on microfossil evidence (error bars represent 95% Posterior Probability (PP) intervals; PP clade support is indicated at nodes).
