# Supplementary material for: The biodiversity hotspot as evolutionary hot-bed: spectacular radiation of Erica in the Cape Floristic Region
Source: BMC Evol Biol. 2016 Sep 17;16:190. doi: 10.1186/s12862-016-0764-3 (PMC5027107; doi:10.1186/s12862-016-0764-3)
Supplement: Additional file 4: Table S2. — Parameter estimates given the three best scoring suboptimal MuSSE models. (DOCX 15 kb) [file 12862_2016_764_MOESM4_ESM.docx]

Table S2: Parameter estimates given the three best scoring suboptimal MuSSE models

| Model 1 |  | λ Cape | Drakensberg | λ Palearctic | λ Madagascar | λ TA | µ Africa | µ Palearctic | transition rate | p |
| --- | --- | --- | --- | --- | --- | --- | --- | --- | --- | --- |
|  | Min. | 0.3990 | 0.08733 | 0.02516 | 0.2619 | 0.08876 | 2.60e-07 | 8.600e-07 | 0.2374 e-03 | -1148 |
|  | 1st Qu. | 0.4606 | 0.35797 | 0.05328 | 0.5168 | 0.26644 | 2.88e-03 | 8.410e-03 | 1.1163 e-03 | -1064 |
|  | Median | 0.4745 | 0.39778 | 0.06353 | 0.5835 | 0.31978 | 6.90e-03 | 1.948e-02 | 1.4031 e-03 | -1062 |
|  | Mean | 0.4749 | 0.40098 | 0.06606 | 0.5883 | 0.32473 | 9.65e-03 | 2.523e-02 | 1.4498 e-03 | -1062 |
|  | 3rd Qu. | 0.4888 | 0.44103 | 0.07602 | 0.6523 | 0.37502 | 1.33e-02 | 3.581e-02 | 1.7341 e-03 | -1061 |
|  | Max. | 0.5879 | 0.65639 | 0.18997 | 1.0694 | 0.74065 | 8.63e-02 | 1.688e-01 | 4.6373 e-03 | -1058 |
|  |  |  |  |  |  |  |  |  |  |  |
| Model 2 |  |  | **λ Africa** | **λ Palearctic** |  |  | **µ Africa** | **µ Palearctic** | **transition rate** | **p** |
|  | Min. |  | 0.4046 | 0.02115 |  |  | 2.20e-07 | 3.880e-06 | 0.1729 e-03 | -1254 |
|  | 1st Qu. |  | 0.4558 | 0.05372 |  |  | 2.91e-03 | 8.860e-03 | 1.1110 e-03 | -1064 |
|  | Median |  | 0.4687 | 0.06381 |  |  | 7.05e-03 | 2.020e-02 | 1.3856 e-03 | -1063 |
|  | Mean |  | 0.4689 | 0.06619 |  |  | 1.01e-02 | 2.554e-02 | 1.4412 e-03 | -1063 |
|  | 3rd Qu. |  | 0.4814 | 0.07613 |  |  | 1.41e-02 | 3.616e-02 | 1.7091 e-03 | -1062 |
|  | Max. |  | 0.5535 | 0.17807 |  |  | 1.06e-01 | 1.616e-01 | 3.72053 e-02 | -1060 |
|  |  |  |  |  |  |  |  |  |  |  |
| Model 3 |  | **λ Cape** | **Drakensberg** | **λ Palearctic** | **λ Madagascar** | **λ TA** | **µ Cape** | **µ non-Cape** | **transition rate** | **p** |
|  | Min. | 0.3865 | 0.1563 | 0.02329 | 0.2810 | 0.1049 | 1.23e-06 | 0.3 e-06 | 0.3027 e-03 | -1245 |
|  | 1st Qu. | 0.4613 | 0.3623 | 0.05161 | 0.5248 | 0.2721 | 3.31e-03 | 6.3469 e-03 | 1.1350 e-03 | -1064 |
|  | Median | 0.4750 | 0.4047 | 0.06145 | 0.5888 | 0.3231 | 7.97e-03 | 1.46527e-02 | 1.4151 e-03 | -1062 |
|  | Mean | 0.4755 | 0.4070 | 0.06322 | 0.5939 | 0.3301 | 1.14e-02 | 1.96317e-02 | 1.4732 e-03 | -1062 |
|  | 3rd Qu. | 0.4893 | 0.4488 | 0.07230 | 0.6586 | 0.3804 | 1.59e-02 | 2.82552e-02 | 1.7396 e-03 | -1061 |
|  | Max. | 0.6191 | 0.6730 | 0.19094 | 1.1509 | 0.7218 | 1.07e-01 | 2.078527 e-01 | 3.08754 e-02 | -1058 |
