# Supplementary material for: The biodiversity hotspot as evolutionary hot-bed: spectacular radiation of Erica in the Cape Floristic Region
Source: BMC Evol Biol. 2016 Sep 17;16:190. doi: 10.1186/s12862-016-0764-3 (PMC5027107; doi:10.1186/s12862-016-0764-3)

Figure S3a
BAMM tree as presented in Fig. 1, including labels for tips and nodes referred to in the text; branches subtending *Erica*, *Calluna* and *Daboecia* are not to scale.
Scale bar is in species per million years

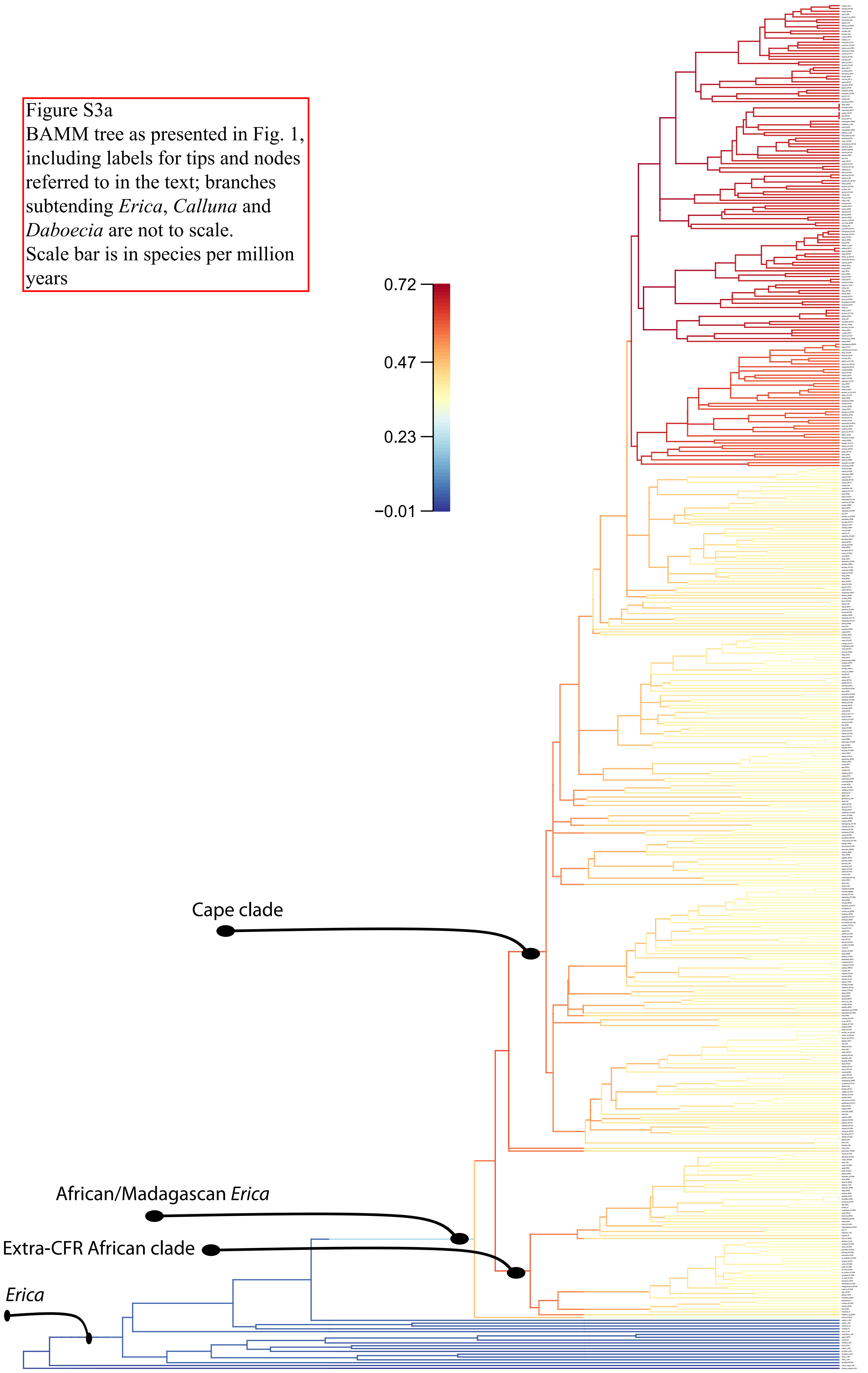

Supplement: Additional file 5: Figures S3. — BAMM diversification rate results: a) BAMM tree as presented in Fig. 1, including labels for tips and nodes referred to in the text; branches subtending Erica, Calluna and Daboecia are not to scale. b) Probabilities of overall numbers of diversification shifts inferred using BAMM. (ZIP 2444 kb) [file 12862_2016_764_MOESM5_ESM.zip › add 6/S3a_Figure_BAMM_best_2016.pdf]
