# Supplementary material for: The biodiversity hotspot as evolutionary hot-bed: spectacular radiation of Erica in the Cape Floristic Region
Source: BMC Evol Biol. 2016 Sep 17;16:190. doi: 10.1186/s12862-016-0764-3 (PMC5027107; doi:10.1186/s12862-016-0764-3)
Supplement: Additional file 6: Table S3. — Summary of BAMM results based on 25 rate-smoothed RAxML bootstrap trees. (DOCX 12 kb) [file 12862_2016_764_MOESM6_ESM.docx]

Table S3: Summary of BAMM results based on 25 rate-smoothed RAxML bootstrap trees.

| **Tree** | **Sum of PP** | **Proportion of configurations showing a shift corresponding to:** | | |
| --- | --- | --- | --- | --- |
|  |  | ***Erica*** | **African/Madagascan clade** | **Within Cape clade** |
| BEST tree | 0.91 | 4/9 | 9/9 | 9/9 |
| Bootstrap 1 | 0.93 | 2/9 | 9/9 | 4/9 |
| 2 | 0.95 | 3/9 | 9/9 | 1/9 |
| 3 | 0.84 | 4/9 | 9/9 | 8/9 |
| 4 | 0.92 | 3/9 | 9/9 | 9/9 |
| 5 | 0.80 | 3/9 | 9/9 | 4/9 |
| 6 | 0.66 | 3/9 | 9/9 | 6/9 |
| 7 | 0.94 | 2/9 | 9/9 | 5/9 |
| 8 | 0.86 | 4/9 | 9/9 | 5/9 |
| 9 | 0.84 | 2/9 | 9/9 | 5/9 |
| 10 | 0.94 | 4/7 | 7/7 | 4/7 |
| 11 | >0.95 | 2/6 | 6/6 | 1/6 |
| 12 | >0.95 | 2/4 | 4/4 | 0/4 |
| 13 | 0.69 | 3/9 | 9/9 | 7/9 |
| 14 | 0.80 | 3/9 | 9/9 | 9/9 |
| 15 | 0.73 | 4/9 | 9/9 | 9/9 |
| 16 | 0.93 | 5/9 | 9/9 | 6/9 |
| 17 | 0.90 | 3/9 | 9/9 | 6/9 |
| 18 | 0.87 | 3/9 | 9/9 | 8/9 |
| 19 | 0.85 | 3/9 | 9/9 | 9/9 |
| 20 | >0.95 | 4/9 | 9/9 | 8/9 |
| 21 | 0.40 | 1/9 | 8/9 | 6/9 |
| 22 | 0.76 | 3/9 | 9/9 | 6/9 |
| 23 | >0.95 | 1/3 | 3/3 | 1/3 |
| 24 | 0.93 | 4/9 | 9/9 | 5/9 |
| 25 | 0.73 | 4/9 | 6/9 | 9/9 |
